# A small secreted protein triggers a TLR2/4-dependent inflammatory response during invasive *Candida albicans* infection

**DOI:** 10.1038/s41467-019-08950-3

**Published:** 2019-03-04

**Authors:** Wenjuan Wang, Zihou Deng, Hongyu Wu, Qun Zhao, Tiantian Li, Wencheng Zhu, Xiongjun Wang, Longhai Tang, Chengshu Wang, Shu-Zhong Cui, Hui Xiao, Jiangye Chen

**Affiliations:** 1 State Key Laboratory of Molecular Biology, Institute of Biochemistry and Cell Biology, University of Chinese Academy of Sciences, Chinese Academy of Sciences, 320 Yue Yang Road, Shanghai, 200031 China; 2 CAS Key Laboratory of Molecular Virology & Immunology, Institut Pasteur of Shanghai, CAS Center for Excellence in Molecular Cell Science, University of Chinese Academy of Sciences, Chinese Academy of Sciences, 320 Yue Yang Road, Shanghai, 200031 China; 3 Suzhou Blood Center, Suzhou, Jiangsu 215000 China; 4 CAS Key Laboratory of Insect Developmental and Evolutionary Biology, Shanghai Institute of Plant Physiology and Ecology, Chinese Academy of Sciences, Shanghai, 200032 China; 5 State Key Laboratory of Respiratory Diseases, Affiliated Cancer Hospital & Institute of Guangzhou Medical University, Guangzhou Medical University, Guangzhou, Guangdong 510095 China

## Abstract

*Candida albicans* can switch from commensal to pathogenic mode, causing mucosal or disseminated candidiasis. The host relies on pattern-recognition receptors including Toll-like receptors (TLRs) and C-type lectin receptors (CLRs) to sense invading fungal pathogens and launch immune defense mechanisms. However, the complex interplay between fungus and host innate immunity remains incompletely understood. Here we report that *C*. *albicans* upregulates expression of a small secreted cysteine-rich protein Sel1 upon encountering limited nitrogen and abundant serum. Sel1 activates NF-κB and MAPK signaling pathways, leading to expression of proinflammatory cytokines and chemokines. Comprehensive genetic and biochemical analyses reveal both TLR2 and TLR4 are required for the recognition of Sel1. Further, *SEL1*-deficient *C*. *albicans* display an impaired immune response in vivo, causing increased morbidity and mortality in a bloodstream infection model. We identify a critical component in the Candida-host interaction that opens a new avenue to tackle Candida infection and inflammation.

## Abstract (web summary)

The interplay between fungal pathogens and the innate immune system remains incompletely understood. Here, Wang et al. show that a small protein secreted by *Candida albicans* induces a TLR2- and TLR4-mediated inflammatory response in a mouse infection model.

## Introduction

Fungal infection has been constantly increasing worldwide, thus posing great threat to human health and life. In particular, invasive candidiasis has become the fourth leading cause of bloodstream infection, annually affecting at least 250,000 people globally, and causing up to 40% mortality in patients^1^. As a commensal microorganism, *Candida albicans* colonizes multiple mucosal sites, including the oral cavity, the gastrointestinal and urogenital tracts, asymptomatically in healthy individuals. In susceptible patients, *C*. *albicans* can enter the bloodstream and cause a frequently fatal disseminated infection. The transformation of *C*. *albicans* from a friendly commensal to a vicious pathogen can be triggered and executed by a variety of factors, such as yeast-to-hyphae transition, adhesin and invasin expression, breach of epithelium or mucosae barrier, as well as primary and acquired immune deficiency^2^. However, the complex interplay between fungal pathogens and the host immune system has just begun to unveil, and further studies aimed to dissect the fungus–host interface will be of considerable significance in promoting future development of therapeutic approaches.

Recent studies from human primary immune deficiency disorders and animal models have led to the discovery of principal innate and adaptive immune components required for the control of local and disseminated fungal infections. By recognizing the fungal cell wall components, TLRs (Toll-like receptors) and CLRs (C-type lectin receptors) play important roles in the initiation of innate immune response for the immediate control of fungal propagation, and the differentiation of CD4^+^ T helper 1 and 17 (Th1 and Th17) effector cells for the later control and long-term memory of fungal infection. Upon detecting the presence of *O*-linked mannosyl proteins^3^, TLR4 engages adaptors MyD88 (myeloid differentiation primary response 88) and TRIF (TIR-domain-containing adapter-inducing interferon-β) to initiate the complex signaling cascades leading to the activation of nuclear factor (NF)-κB and mitogen-activated protein kinases (MAPKs) such as c-Jun N-terminal kinase (JNK), extracellular signal-regulated kinase (ERK), and p38, culminating in the induction of a myriad of proinflammatory cytokines and chemokines, including tumor necrosis factor (TNF), interleukin (IL)-6, IL-1, and chemokine (C-X-C motif) ligand-1/2 (CXCL-1/2). On the other hand, TLR2 primarily recognizes the fungal cell wall component phospholipomannan^4^, and launches MyD88-dependent signaling pathways to activate NF-κB and MAPKs. Additionally, CLR family members dectin-1 and dectin-2/3 mainly recognize β-glucan or α-mannan, respectively. Following ligand engagement, dimerized dectin-1 or dectin-2/3 receptors recruit adaptors SHP-2 or/and FcRγ, leading to the activation of protein kinase Syk, which then promotes the activation of NF-κB and NFAT (nuclear factor of activated T cells), whereby coordinating the induction of TNF, IL-6, and IL-23 in dendritic cells (DCs) and macrophages^5–7^. Recent studies demonstrate that β-glucan-elicited signaling can also trigger mammalian target of rapamycin (mTOR) activation and metabolic–epigenetic reprograming in monocytes and macrophages, which subsequently acquire so-called “trained immunity”, a memory response that enables innate immune cells to mount highly efficient proinflammatory response against recurrent fungal infection^8–10^. In addition, NLRP3- and NLRC4-dependent inflammasomes have also been demonstrated to be crucial for anti-fungal defense through the production of mature IL-1β^11–14^. Interestingly, TLR2 and dectin-1 can work together to recognize fungal cell wall component zymosan, thus demonstrating a paradigm that multiple pattern-recognition receptors (PRRs) can work in synergy in tailoring immune response^15^.

Being an opportunistic pathogen, *C. albicans* has evolved numerous strategies to promote colonization and pathogenesis. Among them, the secreted proteins are drawing increased attention. The hyphae secreted peptide toxin Candidalysin has a critical role in disrupting epithelium membrane and promoting fungal invasion^16,17^. Accordingly, Candidalysin can trigger a stress-related proinflammatory response in the oral and vaginal epithelium, promoting copious production of cytokines IL-1, granulocyte-colony stimulating factor, and TNF^17,18^. Moreover, *C*. *albicans* can secrete various hydrophobic enzymes, notably the secreted aspartic proteinases (Saps). Once secreted into the host niches, Saps can degrade a variety of host proteins, ranging from cell membrane proteins to immune regulators, therefore contributing to fungal virulence^19–21^. Intriguingly, some Saps, particularly Sap2 and Sap6, have also been shown to be able to induce proinflammatory cytokine production through the engagement of TLRs or NLRP3 in vaginitis^22–25^. Similarly, another secreted protein Pra1, known as zinc scavenger^26^, acts through integrin α_M_β_2_ in mediating the recruitment of leukocytes^27,28^. Conceivably, the interaction between *C*. *albicans* secretome and host immune system can be dynamic and context dependent, and it is not surprising that the underlying complexity has just begun to unveil.

To survive a diversity of hostile conditions in the host, plant fungal pathogens often deploy secretory proteins such as effector proteins, elicitins, and cerato-platanins to adapt to specific surroundings, promoting their own growth while interfering with the host defense mechanism^29–31^. While the vast majority of fungal-secreted proteins have been demonstrated to function as virulence factors by suppressing the plant defense mechanism, some of them can also be recognized by the host surveillance systems and act as elicitors of host defense response, hence playing an important role in host–fungus interaction^32–35^. Notably, most of these secreted proteins shared common characteristics, such as being of small size, rich in cysteine content, and with a signal peptide, as demonstrated by previous studies on small secreted cysteine-rich proteins (SCPs) mining in plant fungal pathogens^29,36–39^.

In this study, we develop a platform combining in silica genomic search with fungus-macrophage interaction assay, and reveal the existence of SCPs in human fungal pathogen *C*. *albicans* accordingly. Among the putative *Candida* SCPs, we demonstrate Sel1 (previously known as Coi1, Orf19.5063) as the most abundantly induced SCP capable of shaping host immune response and the severity of fungal systemic infection. Notably, our results suggest that Sel1 might function as a novel fungus-derived pattern-associated molecular pattern (PAMP) for mammalian TLR2 and TLR4, whereby eliciting robust proinflammatory response in macrophages, DCs, and monocytes. Collectively, our results shed light on the complex host immune recognition of fungal pathogens, and open a new avenue for the development of treatment on fungus-induced sepsis.

## Results

### Sel1 is an immunostimulatory SCP in *C*. *albicans*

**Fig. 1:**
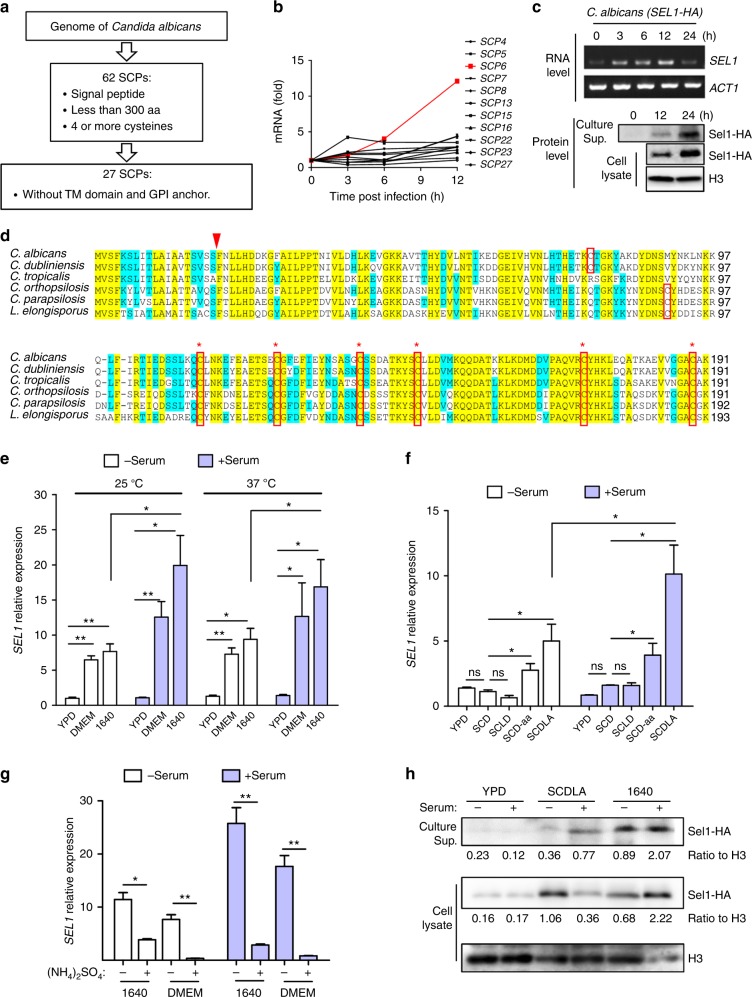
Screening and validation of Sel1 as a SCP of *C*. *albicans*. **a** Schematic presentation of in silico screening of SCPs in *C*. *albicans*. **b** *C*. *albicans* SC5314 cells were incubated with Raw 264.7 cells, and the expression levels of SCPs were analyzed by real-time quantitative PCR (qRT-PCR). **c** Bone marrow-derived macrophages (BMDMs) were infected by *C*. *albicans* harboring *SEL1-HA*. The culture supernatants (Culture Sup.) were collected, centrifuged, and filtered with 0.22 μm column prior to western blotting, while the remaining BMDMs and fungal cells were washed with water and harvested by centrifugation for RNA or protein preparation, respectively. *SEL1 (SCP6)* RNA levels (top panels) and Sel1-HA (Scp6-HA) protein levels (bottom panels) were assessed by RT-PCR or western blotting. **d** Sequence alignment of Sel1 homologs in *Candida* species. N-terminal signal peptide is indicated by red arrow. The cysteine residues are shown in red box, and the conserved cysteines are marked by red asterisks. **e**–**g** *C*. *albicans* cells grown in various media (6 h) as indicated were harvested and *SEL1* expression was analyzed by qRT-PCR at 25 °C for **f**, **g**. YPD yeast extract peptone dextrose medium, DMEM Dulbecco’s modified Eagle's medium, 1640 Roswell Park Memorial Institute 1640 medium, SCD synthetic complete dextrose, SCLD SCD medium with low dextrose, SCD-aa SCD medium without amino acids, SCDLA SCD medium with low ammonium. The ingredients of each medium can be found in the Methods section. **h** *C*. *albicans* (*SEL1-HA*) were grown in various media as indicated for 12 h. The culture supernatants (Culture Sup.) were collected as in **c**, whereas the fungal cells were collected and lysed for protein lysates. The Sel1-HA proteins in the supernatants and cell lysates were detected by western blotting and quantified over histone H3 (loading control). Data represent the average expression levels of two independent experiments (**e**–**g**). Bars, mean ± SEM. Data are representative of at least two (**b**, **c**, **h**) independent experiments each with similar results. Data relative to RNA level in the un-induced *C*. *albicans* cells (**b**, **d**–**f**); *p < 0.05, **p < 0.01, ns: no significance, by Student’s *t*-test

While small secreted cysteine-rich featured proteins are widely involved in fungus–plant interaction, such proteins have not been reported in human fungal pathogens yet. In this regard, we sought to identify putative SCPs in the most prevalent human fungal pathogen *C*. *albicans*, and search for candidates with the potential of shaping host immune response. Through preliminary in silica screening (Fig. 1a), we identified 27 candidate SCP-encoding genes within the genome of *C*. *albicans*. These genes presumably encode small proteins (<300 amino acids) comprising a signal peptide and at least four cysteines. We also excluded the genes encoding proteins with putative transmembrane domain or glycosylphosphatidylinositol-anchor, which are characteristics typically associated with cell membrane or cell wall proteins^38–40^ (Fig. 1a). Next, we examined the expression patterns of these SCP-encoding genes during *C*. *albicans* infection, in particular upon encountering immune cells. Remarkably, the expression levels of 11 out of 27 SCP-encoding genes were elevated in *C*. *albicans* incubated with murine macrophage RAW 264.7 cells, with *SCP6* showing the highest induction among them (Fig. 1b). Further, *SCP6* expression was also markedly induced in *C*. *albicans* following incubation with mouse bone marrow-derived macrophages (BMDMs) (Fig. 1c, Supplementary Figure 1a). It is important to note that Scp6 proteins were evidently detected in the culture media of BMDMs infected with *C*. *albicans* strain expressing hemagglutinin (HA)-tagged Scp6 (Fig. 1c). As the culture media were depleted of macrophages and fungal cells, the detection of Scp6-HA in the media suggested that *C*. *albicans*-expressed Scp6 proteins were secreted into the BMDM culture media (Fig. 1c), thus confirming the induction and secretion of Scp6 during macrophage infection. The gene *SCP6* (*COI1*, *ORF19*.*5063*) encodes a small protein composed of 191 amino acids, including 7 cysteine residues and a signal peptide at its N terminus (Fig. 1d). The Orf19.5063 was originally designated as Coi1 (Ciclopirox Olamine Induced) by Sigle et al.^41^ based on mass spectrometric analysis. Considering its newly identified physiological function which will be described in detail below, we propose to rename Orf19.5063 as Sel1 (Secreted Elicitor 1) hereafter.

The prominent *SEL1* expression was not only detected in *C*. *albicans* incubated with BMDMs, but also in *C*. *albicans* cultured with RPMI-1640 (10% fetal bovine serum (FBS)) medium (Supplementary Figure 1a), implicating important roles for culture media and growth conditions in *SEL1* induction. Unlike Candidalysin, *SEL1* expression was not affected by yeast-to-hyphae transition, thus showing similar expression levels under both yeast growth (yeast extract peptone dextrose (YPD), 25 °C) and hyphae induction (YPD with serum, 37 °C) (Supplementary Figure 1b, c). In contrast, *SEL1* expression was greatly induced by synthetic mammalian cell culture media Dulbecco’s modified Eagle's medium (DMEM) and RPMI-1640, an effect further enhanced by serum rather than high temperature (Fig. 1e). Since synthetic media DMEM and RPMI-1640, but not rich media YPD, demonstrated strong effect on *SEL1* induction (Fig. 1e), we subsequently interrogated the role of nutrients, such as carbon, amino acids, and nitrogen in this process. *C*. *albicans* cultured in the synthetic complete medium with 2% glucose (synthetic complete dextrose (SCD)) expressed similar amounts of *SEL1* to those cultured in YPD (Fig. 1f). Although lowering glucose (0.1%, SCLD) (Fig. 1f) or substituting glucose with other carbon sources including *N*-acetyl-glucosamine, lactate, and glycerol (Supplementary Figure 1d) failed to elevate *SEL1* expression, depleting amino acids (SCD without amino acids, SCD-aa) or lowering nitrogen source (SCD with low ammonium sulfate (SCDLA)) was able to elevate *SEL1* expression, especially in the presence of serum (Fig. 1f). In addition, *SEL1* expression was also highly induced in nitrogen limited Lee’s medium and Lee’s-LA medium (with low ammonium sulfate) in the presence of serum (Supplementary Figure 1e). In contrast, increasing ammonium amount in DMEM or RPMI-1640 medium (supplement with 37 mM ammonium sulfate) resulted in pronounced decrease in *SEL1* expression, even overriding the positive effect of serum (Fig. 1g). Therefore, nitrogen abundance appeared to play a pivotal role in *SEL1* expression. Consistently, we also detected higher amounts of Sel1-HA proteins in *C*. *albicans* cultured in SCDLA and RPMI-1640 media than in YPD. Remarkably, by assessing the Sel1-HA secreted into the supernatants, we found that nitrogen starvation and serum are important in facilitating the secretion of Sel1 (Fig. 1h). These results demonstrate nitrogen limitation and serum presence act in synergy in facilitating Sel1 expression and secretion.

**Fig. 2:**
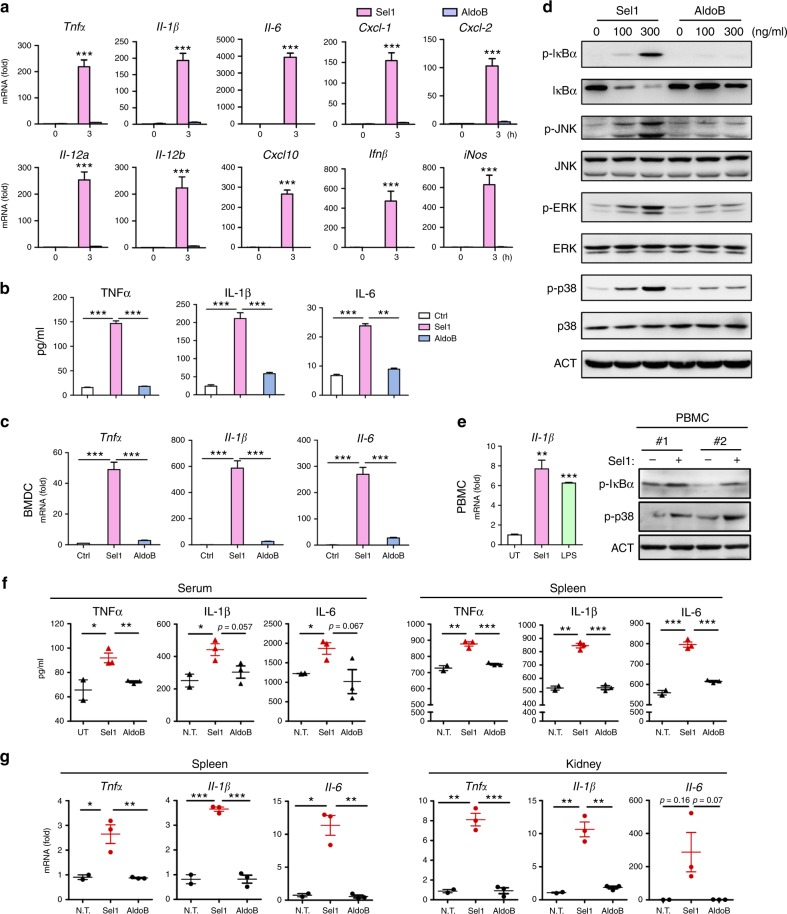
Sel1 induces proinflammatory responses in immune cells. **a**, **b** Bone marrow-derived macrophages (BMDMs) were stimulated with purified recombinant Sel1 and AldoB proteins (300 ng/ml) for 3 h, and the induction of proinflammatory genes was measured by real-time quantitative PCR (qRT-PCR) (**a**) or enzyme-linked immunosorbent assay (ELISA) (**b**), respectively. **c** Bone marrow- derived dendritic cells (BMDCs) were stimulated with Sel1 or AldoB proteins (300 ng/ml) for 3 h, and the cytokine response was assessed by qRT-PCR. **d** BMDMs were stimulated with various amounts of Sel1 and AldoB proteins for 30 min, and the activation of nuclear factor (NF)-κB and mitogen-activated protein kinases (MAPKs) were examined by western blotting. **e** Peripheral blood mononuclear cells (PBMCs) were stimulated with Sel1 (300 ng/ml) or lipopolysaccharide (LPS; 100 ng/ml) for 3 h, and *Il-1β* production was measured by qRT-PCR (left), or PBMCs from two individuals were stimulated with Sel1 (300 ng/ml, 30 min) and the signaling pathways were analyzed by western blotting (right). **f**, **g** Wild-type C57BL/6 mice were injected with purified Sel1 or AldoB proteins, respectively, and 6 h later, the serum and tissues were obtained for cytokine detection (6–8 weeks old, male, 150 μg protein/mouse, *n* = 3). Tumor necrosis factor-α (TNFα), interleukin (IL)-1β, and IL-6 proteins in serum (left) and spleen (right) was measured by ELISA (**f**). *Tnfα*, *Il-1β*, and *Il-6* mRNA levels in spleen (left) and kidney (right) were assessed by qRT-PCR (**g**). Data represent the average expression levels of each gene from at least two (**a**–**c**, **e**–**g**) independent experiments. Data are representative of at least three (**d**) independent experiments with similar results. Bars, mean ± SEM; *p < 0.05, **p < 0.01, ***p < 0.001, by Student’s *t*-test

Next, we expressed and purified recombinant His-tagged Sel1 proteins from *Escherichia* *coli* by fast protein liquid chromatography, followed by endotoxin removal (Supplementary Figure 1f). Remarkably, stimulation with Sel1 protein was able to elicit prominent proinflammatory response in BMDMs (Fig. 2a, b). Real-time quantitative PCR (qRT-PCR) analyses revealed marked induction of proinflammatory genes, such as *Tnf*, *Il1β*, *Il6*, *Cxcl1*, *Cxcl2*, *Il12a*, *Il12b*, *Cxcl10*, *Ifnβ*, and *iNos* by Sel1 stimulation (Fig. 2a). By enzyme-linked immunosorbent assay (ELISA), we also detected copious production and secretion of TNF, IL-1β, and IL-6 by Sel1-stimulated BMDMs (Fig. 2b). In contrast, His-AldoB, a control recombinant protein prepared and treated the same as His-Sel1, failed to elicit meaningful proinflammatory response in BMDMs, supporting the specific effect of Sel1 (Fig. 2a, b). Moreover, Sel1 stimulation also elicited robust proinflammatory response in bone marrow-derived dendritic cells (BMDCs) (Fig. 2c), leading to prominent induction of *Tnf*, *Il1β*, and *Il6* expression.

We then investigated the molecular mechanism by which Sel1 exerts its proinflammatory effect. By western blotting, it was evident that Sel1 induced the phosphorylation and degradation of IκBα in BMDMs, indicative of NF-κB activation (Fig. 2d). Additionally, Sel1 stimulation also promoted the phosphorylation and activation of MAPKs, including JNK, ERK, and p38 (Fig. 2d). To extend above findings to humans, Sel1 stimulation also triggered the activation of NF-κB and p38 in human peripheral blood mononuclear cells (PBMCs), leading to the induction of Il1β in human monocytes (Fig. 2e). The above data prompted us to ask whether Sel1 may be capable of triggering inflammatory response in vivo. To this end, we administered Sel1 proteins into mice through tail vein injection and analyzed cytokine production in 6 h (Fig. 2f, g). Remarkably, Sel1 induced considerable production of TNF, IL-1β, and IL-6 in both mouse sera and spleens (Fig. 2f). Consistently, qRT-PCR analyses also revealed potent inflammatory responses elicited by Sel1 in the spleen and kidney (Fig. 2g). These results collectively demonstrate a remarkable proinflammatory nature for Sel1. Collectively, these data indicate *Candida*-encoded Sel1 as a SCP with unique immune eliciting capacity and being upregulated under hostile conditions such as nitrogen limitation and serum stimulation.

### Sel1 activates TLRs in eliciting proinflammatory response

**Fig. 3:**
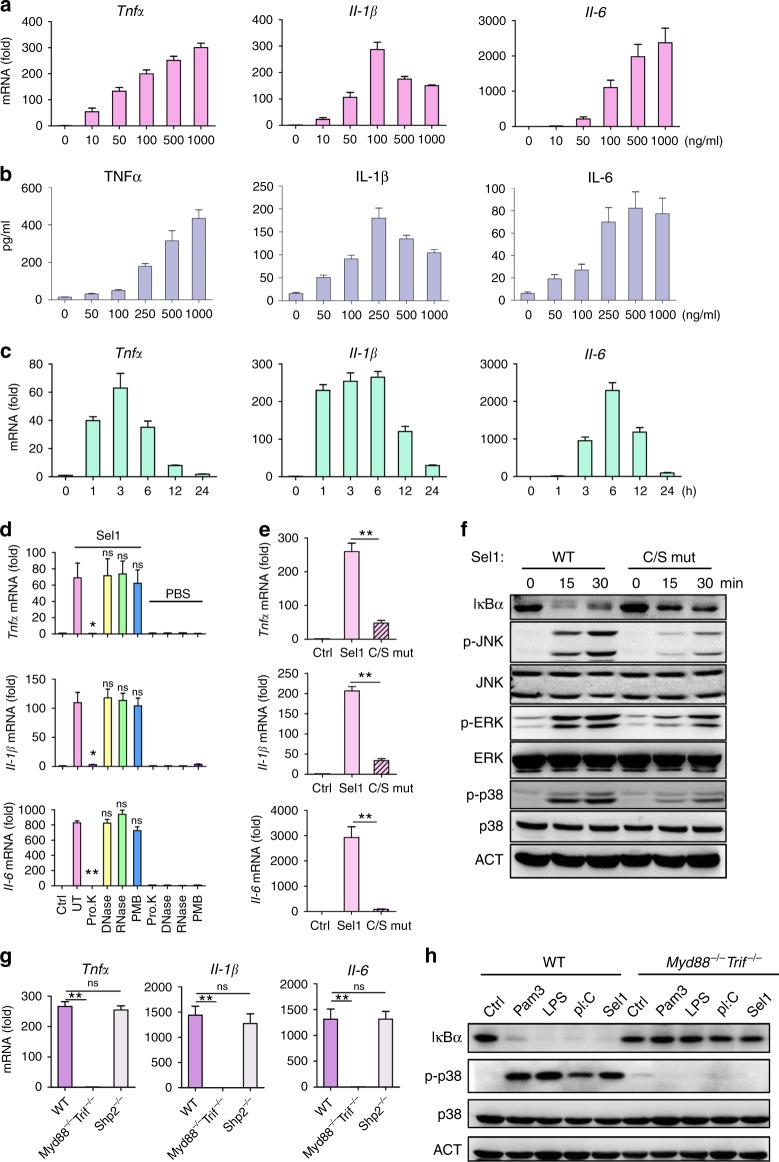
Sel1 activates MyD88/Trif-dependent inflammatory responses. **a**, **b** Bone marrow-derived macrophages (BMDMs) were stimulated with different doses of Sel1 for 3 h (**a**) or 24 h (**b**), and the cytokine production was assessed by real-time quantitative PCR (qRT-PCR) (**a**) or by enzyme-linked immunosorbent assay (ELISA) (**b**), respectively. **c** BMDMs were stimulated with 300 ng/ml of Sel1 for various times and the cytokine production was assessed by qRT-PCR. **d** The Sel1 proteins were either untreated (UT) or treated with proteinase K, DNase, RNase or PMB, prior to stimulation. BMDMs were stimulated with pretreated Sel1 for 3 h, and the cytokines were assessed by qRT-PCR. **e**, **f** Purified wild-type (WT) or Cys to Ser mutant (C/S mut) Sel1 proteins were used for BMDM stimulation. The cytokine production (**e**) and signaling pathways (**f**) were analyzed by qRT-PCR or western blotting, respectively. **g** The WT, *Myd88*^−/−^*Trif*^−/−^, or *Shp2*^−/−^ BMDMs were stimulated with Sel1, and cytokine production was quantified by qRT-PCR. **h** WT, *Myd88*^−/−^*Trif*^−/−^ BMDMs were stimulated with the indicated stimuli for 30 min, and signaling pathways were analyzed by western blotting. Pam3C Pam3CSK4, LPS lipopolysaccharide, pI:C polyinosinic:polycytidylic acid. Data represent the average expression levels of each gene from three (**a**–**e**, **g**) independent experiments. Bars, mean ± SEM. Data are representative of at least three (**f**, **h**) independent experiments with similar results; *p < 0.05, **p < 0.01, ns: no significance, by Student’s *t*-test

Subsequently, we investigated the kinetics and dose effect of Sel1 with respect to inflammatory response. It is noteworthy that Sel1 acted in a dose-dependent manner to promote *Tnf*, *Il1β*, and *Il6* expression in BMDMs. While Sel1 was able to trigger *Tnf* and *Il1β* expression at concentrations as low as 10 ng/ml, its optimal dosage for inflammatory response seemed to fall into the range of 250–500 ng/ml (Fig. 3a, b and Supplementary Figure 2a). Strikingly, Sel1 was able to induce notable p38 activation in 15 min after BMDM stimulation (Supplementary Figure 2b), corroborating with robust induction of *Tnf* and *Il1β* mRNAs in 1 h, and *Il6* mRNAs in 3 h, respectively (Fig. 3c). In accordance, Sel1 induced cytokine production in BMDCs in a dose- and time-dependent manner as well (Supplementary Figure 2c, d). These data indicate that Sel1 can elicit proinflammatory response in a fast and dose-dependent way. Importantly, pretreating the purified recombinant Sel1 proteins with protease K, but not DNase I or RNase A, abrogated its immunostimulatory effect, resulting in blunted proinflammatory gene expression and p38 phosphorylation in BMDMs (Fig. 3d and Supplementary Figure 2e), thus ruling out possible contamination with DNA or RNA in our Sel1 proteins. Likewise, pretreatment with polymyxin B (PMB), an inhibitor of lipopolysaccharide (LPS), also failed to compromise the proinflammatory effect of Sel1, albeit it effectively blocked LPS effect (Fig. 3d and Supplementary Figure 2e). Together, these results further reinforced the notion that *Candida*-derived Sel1 may function as a protein agonist capable of triggering proinflammatory response in the host.

Besides its cysteine-rich feature, database search failed to reveal any characterized motifs or domains for Sel1, and we therefore investigated the role of cysteines in Sel1 function by mutagenesis. Remarkably, replacing all 7 cysteine residues with serine markedly impaired the proinflammatory function of Sel1 (Fig. 3e, f). The observation that the Cys-to-Ser mutant of Sel1 was severely compromised in the activation of NF-κB and MAPKs underscored an indispensable role for cysteines in Sel1 function, one of the characteristics well demonstrated for plant fungal SCPs.

As TLRs and CLRs are the two major PRRs responsible for fungus-induced proinflammatory response, we then asked which family of receptors might be involved in the Sel1 effect. While SHP-2 has been shown to be critical for zymosan- and mannan-induced cytokine responses^5^, *Shp-2*^−/−^ BMDMs responded normally to Sel1 stimulation (Fig. 3g). Moreover, unlike zymosan-depleted, Sel1 stimulation failed to promote Syk phosphorylation in BMDMs (Supplementary Figure 2f). Therefore, it seemed unlikely for Sel1 to act through CLRs, as dectin-1, dectin-2/3, and Mincle all signal through SHP-2 and Syk. On the other hand, Sel1-induced cytokine response was blunted in BMDMs deficient of both MyD88 and TRIF (Fig. 3g and Supplementary Figure 2g). Moreover, MyD88/TRIF-double knockout (DKO) BMDMs also failed to exhibit NF-κB or MAPK activation following Sel1 stimulation (Fig. 3h). These results suggest a scenario that Sel1 might engage TLRs rather than CLRs for the proinflammatory response.

### Sel1 activates host immune response through TLR2 and TLR4

**Fig. 4:**
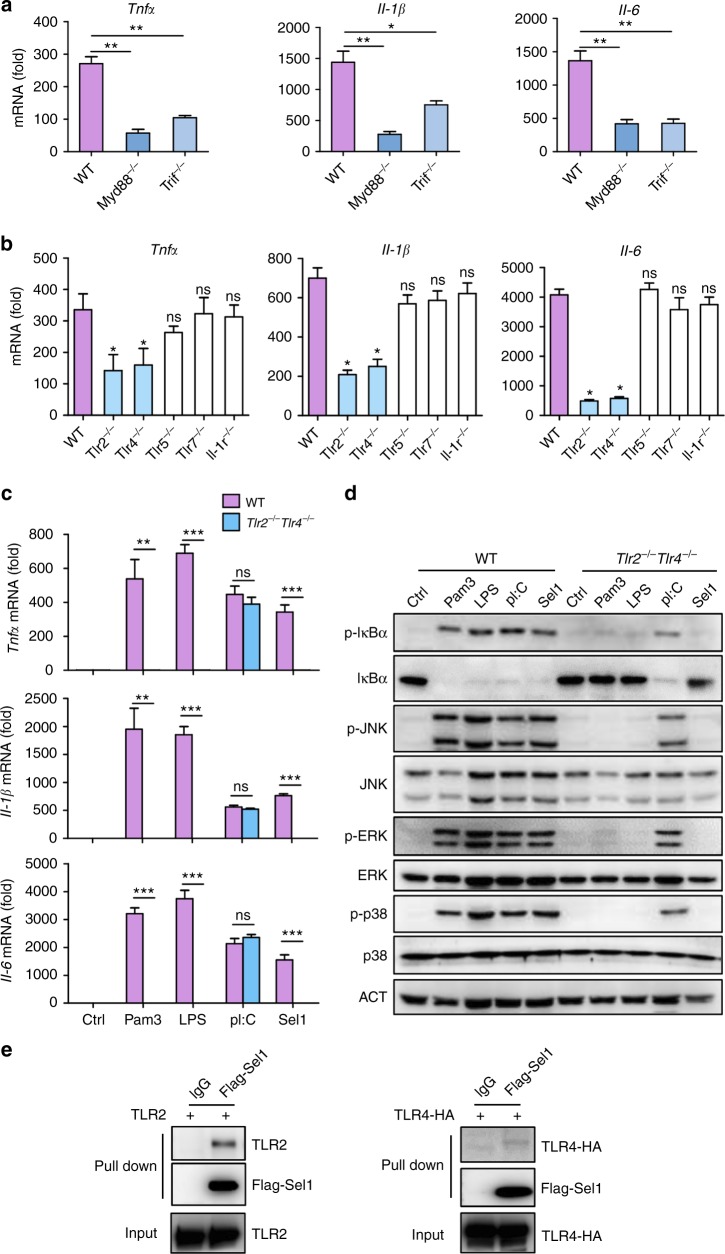
TLR2 and TLR4, but not other TLRs, are involved in Sel1 recognition. **a**, **b** A panel of TLR adaptor- or receptor-deficient C57BL/6 bone marrow-derived macrophages (BMDMs) were stimulated with Sel1, and cytokine production was analyzed by real-time quantitative PCR (qRT-PCR). **c**, **d** The wild-type (WT) and *Tlr2*^−/−^*Tlr4*^−/−^ BMDMs were stimulated with Sel1 and various TLR ligands, and the cytokine production (**c**) or signaling pathways (**d**) were analyzed by qRT-PCR or western blotting, respectively. **e** Western blotting analysis of proteins pulled down from lysates of HEK293T cells transiently transfected with plasmids expressing TLR2 or TLR4-HA, respectively. IgG flag antibody-conjugated agarose only, Flag-Sel1 flag antibody conjugated agarose bound with Flag-Sel1 protein. Data represent the average expression levels of genes from three independent experiments (**a**–**c**). Bars, mean ± SEM. Data are representative of at least two independent experiments, and similar results were obtained (**d**, **e**); *p < 0.05, **p < 0.01, ***p < 0.001, ns: no significance, by Student’s *t*-test

As MyD88/TRIF-DKO macrophages are unresponsive to all the TLRs and IL-1R signals, we further assessed the relative contribution of MyD88 and TRIF in Sel1 proinflammatory response. While MyD88/TRIF double deficiency was able to abolish Sel1 effect (Fig. 3g, h), MyD88 or TRIF deficiency alone only partially impaired Sel1-induced cytokines in BMDMs (Fig. 4a and Supplementary Figure 3a, b), suggesting Sel1 effect might be contingent on both MyD88 and TRIF. Among the TLRs known to be involved in *Candida*-specific immune response, TLR4 engages both MyD88 and TRIF^42^, and we therefore tested the involvement of TLR4 in this process subsequently. Surprisingly, although genetic ablation of TLR4 did result in marked reduction of cytokines, Sel1 still managed to trigger significant cytokine responses in TLR4-deficient BMDMs (Fig. 4b). Consistently, western blotting revealed diminished JNK and ERK activation, but normal NF-κB and p38 activation, in Sel1-stimulated TLR4-deficient BMDMs (Supplementary Figure 3c). These data collectively not only demonstrate a critical role for TLR4 in Sel1 effect, but also suggest the involvement of other TLRs.

By examining a panel of BMDMs with respective TLR deficiency, we found that TLR2, but not TLR5 or TLR7, was also partly required for Sel1-induced proinflammatory responses (Fig. 4b and Supplementary Figure 3d). On the other hand, IL-1R deficiency did not have any significant impact on Sel1 response, thus excluding the involvement of IL-1R signaling in this effect (Fig. 4b).

To further interrogate a possible redundant role for TLR2 and TLR4 in Sel1 response, we used both genetic ablation and chemical blockade to achieve TLR2/TLR4 double deficiency. Remarkably, Sel1-induced cytokine production and signaling events, including the activation of NF-κB and MAPKs, were all abrogated in TLR2/TLR4-DKO BMDMs (Fig. 4c, d). Consistently, when applying chemical inhibitors of TLR4 (TAK242 or ATL1) to TLR2-deficient BMDMs to achieve concomitant TLR2 and TLR4 blockade, Sel1-elicited cytokines and signaling events were also unanimously abolished (Supplementary Figure 3e, f). To test whether Sel1 can physically interact with TLR2 and TLR4, we performed in vitro pull-down assay accordingly. The Flag-tagged Sel1 lacking the N-terminal signal peptide was overexpressed in 293T cells and then immunoprecipitated with anti-FLAG M2 beads. By incubating with 293T cell lysates containing either overexpressed TLR2 or TLR4, the Flag beads bound with Sel1 proteins pulled down both TLR2 and TLR4, whereas the control beads failed to do so (Fig. 4e). These results suggest that Sel1 might be able to form complexes with either TLR2 or TLR4 in the induction of proinflammatory responses.

### *SEL1* regulates the immune response and pathogenesis

**Fig. 5:**
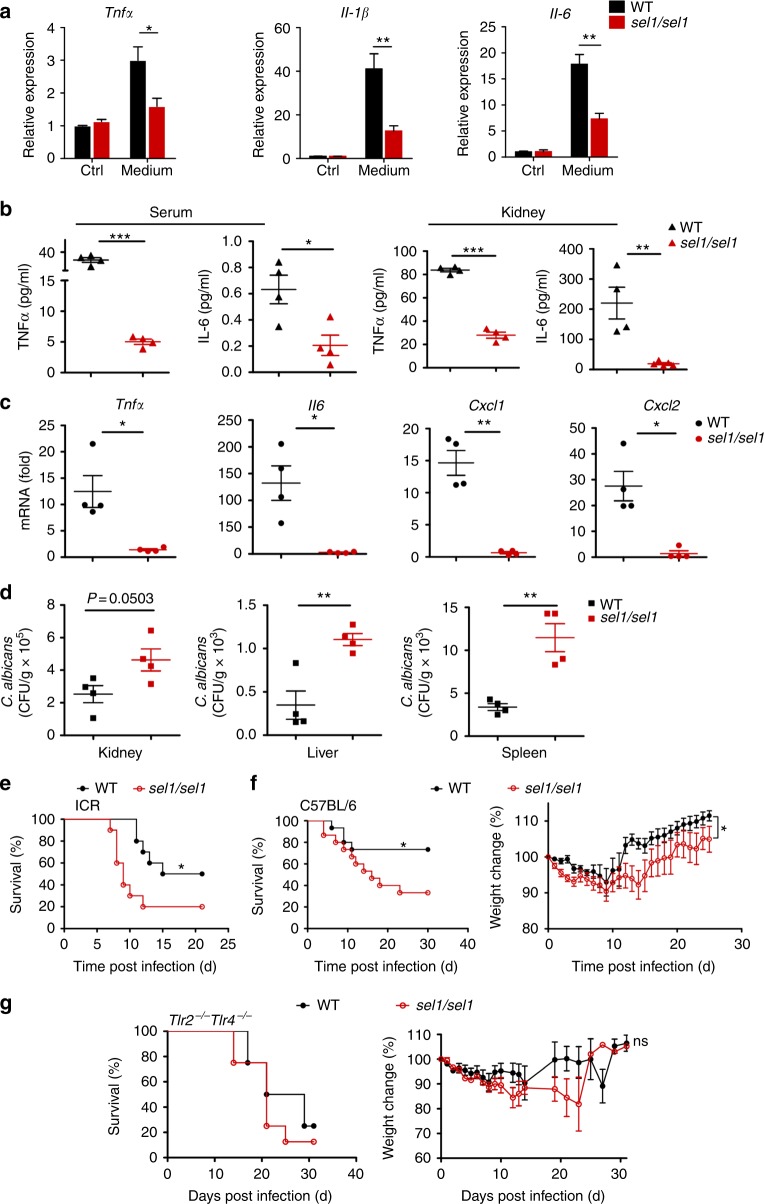
*C*. *albicans*-derived Sel1 contributes to host immune response and candidiasis. **a** Wild-type (WT) or *sel1/sel1* mutant *C*. *albicans* were cultured in RPMI-1640 medium with 10% fetal bovine serum (FBS) for 12 h, and the culture supernatants were collected by filtration with 0.22 μm filter. Filtered supernatants were used for bone marrow-derived macrophage (BMDM) stimulation, and the induction of cytokines was analyzed by real-time quantitative PCR (qRT-PCR). **b**–**d** ICR mice were intravenously infected with WT or *sel1/sel1 C*. *albicans* (male mice of 18–21 g weight were inoculated with 1 × 10^5^ CA cells/mouse; *n* = 4 for **b**–**d**, and *n* = 10 for **e**). Tumor necrosis factor-α (TNFα) and interleukin-6 (IL-6) protein levels in serum (left) and kidney (right) were measured by enzyme-linked immunosorbent assay (ELISA) on day 5 post infection (dpi 5) (**b**). The mRNA levels of cytokines and chemokines in kidneys were detected (dpi 5) by qRT-PCR (**c**). Recoverable fungal colony-forming units (CFUs) in infected mouse tissues (kidney, liver, and spleen, dpi 5) were quantified, and results were presented as CFU per gram of the tissue (**d**). **e** Percentage of survival of ICR mice infected intravenously. **f** C57BL/6 mice (male, 6 weeks, *n* = 15, 1 × 10^5^ cells/mouse) were infected with WT or *sel1/sel1* mutant *C*. *albicans*. Survival percentages (left) and weight changes shown as percentages (right) were analyzed. **g** *Tlr2*^−/−^*Tlr4*^−/−^ mice (male, 6 weeks, *n* = 8, 1 × 10^5^ CA cells/mouse) were infected with WT or *sel1/sel1 C*. *albicans*, and the survival percentages (left) and weight changes (right) are shown. Data represent the average expression levels of genes from three independent experiments (**a**). Bars, mean ± SEM; *p < 0.05, **p < 0.01, ***p < 0.001, by Student’s *t*-test (**b**–**d**) or by log-rank test (**e**–**g**)

Subsequently, we tested whether *C*. *albicans*-derived Sel1 can be involved in the regulation of host immune response in vivo. To this end, we generated a gene-targeted mutant *sel1/sel1 C*. *albicans* strain which does not express Sel1. While the supernatants collected from wild-type *C*. *albicans* cultured under RPMI-1640 medium with 10% serum were able to elicit potent cytokine response when added into BMDMs (Fig. 5a), the supernatants from *sel1/sel1* mutant fungal culture induced much less expression of *Tnf*, *Il-1β,* and *Il6* in BMDMs (Fig. 5a), further implicating that Sel1 may contribute to *C*. *albicans*-induced immune response.

To test the role of Sel1 in invasive fungal infection, wild-type and *sel1*/*sel1* mutant *C*. *albicans* were intravenously injected into ICR mice, followed by the analyses of host immune responses 5 days after infection. Compared to the wild-type strain, *SEL1-*deficient *C*. *albicans* triggered less amounts of TNF and IL-6 in the sera and kidneys of infected mice (Fig. 5b). Moreover, decreased chemokines *Cxcl1* and *Cxcl2*, along with cytokines *Tnf* and *Il6*, were also associated with *sel1*/*sel1 C*. *albicans* infection (Fig. 5c), revealing an important role for Sel1 in *C*. *albicans*-induced immune response. Corresponding to impaired immune response upon *sel1*/*sel1* infection, mice infected with the mutant *C*. *albicans* exhibited heightened fungal burdens in multiple organs, including the kidney, liver, and spleen (Fig. 5d). Consequently, mice infected with *sel1*/*sel1* mutant *C*. *albicans* suffered greater mortality than those infected with the wild-type strain at both 1 × 10^5^ cells/mouse and 5 × 10^5^ cells/mouse inoculum sizes (Fig. 5e, Supplementary Figure 4a). In addition to ICR mice, C57BL/6 mice systemically infected with *sel1*/*sel1* strain also suffered severer morbidity and mortality (Fig. 5f).

To test the role of Sel1-TLR2/4 axis in fungal infection, we infected the *Tlr2*/*Tlr4* double knockout mice with wild-type and *sel1*/*sel1* mutant *C*. *albicans*. Unlike the wild-type mice, the *Tlr2*/*Tlr4-*DKO mice showed compared mortalities to infection with either *sel1*/*sel1* mutant or wild-type *C*. *albicans* (Fig. 5g, Supplementary Figure 4b), supporting an important role for Sel1 in the regulation of host immune defense to fungal infection.

**Fig. 6:**
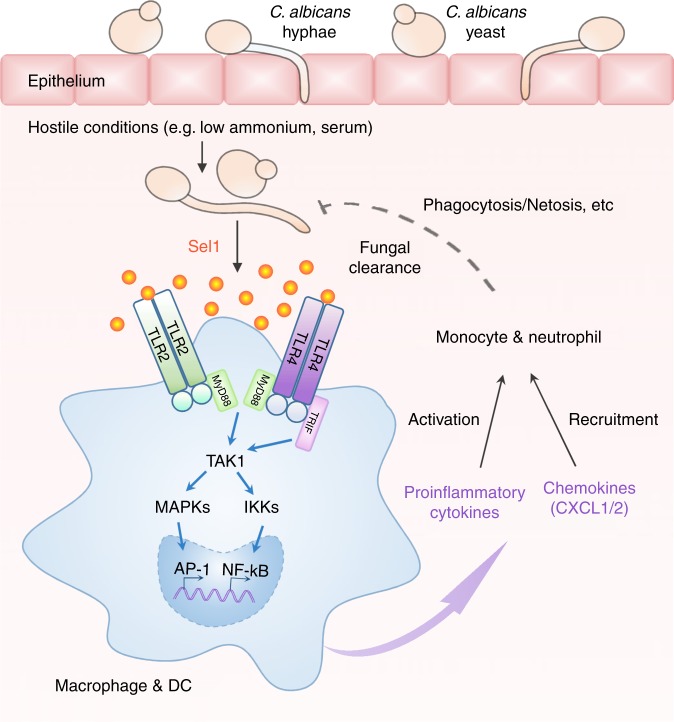
Sel1 represents a novel fungus-derived PAMP for host immune response. Upon invasive infection, *C*. *albicans* encounter the hostile conditions, such as low ammonium and rich serum, which are widely present in the bloodstream and various tissue environments, therefore elevating Sel1 expression/secretion accordingly. Following its secretion, Sel1 can be sensed by both Toll-like receptors 2 and 4 (TLR2 and TLR4), which are abundantly expressed in macrophages and dendritic cells (DCs), leading to the activation of IKK/NF-κB and MAPK/AP-1 (mitogen-activated protein kinase/activator protein 1) pathways, triggering the production of cytokines such as tumor necrosis factor (TNF), interleukin (IL)-6, and IL-1, and chemokines CXCL1 and CXCL2 (chemokine (C-X-C motif) ligands 1 and 2) to recruit and activate neutrophils and monocytes for fungal clearance

It is important to note that the *sel1*/*sel1* mutant not only grew similarly to the wild-type *C*. *albicans* (Supplementary Figure 5a), but also conducted yeast-to-hyphae transition normally in a variety of assays (Supplementary Figure 5b–e). Firstly, both wild-type and *sel1*/*sel1* yeast cells developed mature filaments under hyphae-inducing condition (Supplementary Figure 5b), both of which were also able to revert to yeast normally (Supplementary Figure 5c). In addition, *sel1*/*sel1* strain did not show any defect in yeast-to-hyphae transition under embedded solid YPS media (Supplementary Figure 5d) or during BMDM infection (Supplementary Figure 5e). These results indicate that Sel1 does not affect the growth or yeast-to-hyphae transition of *C*. *albicans*. Furthermore, when incubated with BMDMs, *sel1*/*sel1* cells were phagocytized and killed by macrophages as effectively as the wild-type fungal cells (Supplementary Figure 5f, g). In addition, both wild-type and *sel1*/*sel1 C*. *albicans* led to macrophage lysis to the same extent in vitro (Supplementary Figure 5h). These data collectively suggest that Sel1 impact on immune response might be a central aspect of *C*. *albicans* invasion and pathology during systemic infection (Fig. 6).

## Discussion

*C*. *albicans* is the most common opportunistic human fungal pathogen and can thrive as commensal or pathogen, largely attributable to its remarkable adaptability to various host environmental niches. Upon pathogenic transformation, *C*. *albicans* can cause either superficial infection on mucosae or invasive infection to the bloodstream. Conceivably, *C*. *albicans* alters its morphology and physiology to adapt to such diverse environmental niches, as well as coping with distinct immune defense mechanisms^43,44^. Through the combination of bioinformatical mining and gene expression profiling, we firstly identified a small secreted cysteine-rich protein in *C*. *albicans*, designated Sel1. We reported that *C*. *albicans* upregulate the expression of Sel1 upon encountering nitrogen limitation and serum, two hallmarks of human bloodstream, leading to marked proinflammatory response. We also provided compelling genetic and biochemical evidence indicating that Sel1 may serve as a fungal PAMP for both TLR2 and TLR4, providing the first paradigm that TLR2 and TLR4 may recognize the same PAMP for host defense.

The *SEL1* homologs are only present in a subset of *Candida* species, including *Candida albicans*, *Candida dubliniensis*, *Candida orthopsilosis*, *Candida tropicalis*, *Candida parapsilosis*, and *Lodderomyces elongisporus* (Fig. 1d), but absent in other human fungal pathogens, such as *Aspergillus* and *Cryptococcus* strains, or some closely related fungal species, like *Saccharomyces cerevisiae*. Notably, all six *SEL1*-encoding *Candida* species have been implicated in human bloodstream infection^45^. During disseminated candidiasis, *C. albicans* become exposed to bloodstream, an environment with low ammonium, but abundant serum, and our data strongly suggest that this kind of environment can act as a trigger to promote robust Sel1 expression and secretion. Upon induction by the bloodstream environment, Sel1 can be recognized by the host surveillance system and provoke host defense (Fig. 6). Therefore, the existence of Sel1 and its homologs in a subset of opportunistic fungal pathogens can be of evolutionary importance.

Among the fungal kingdom, the SCPs have been intensively studied in phytopathogenic microorganisms, and most of which have been defined as effectors. Though known for their ability to suppress host defense response, the fungal effectors can also be recognized by host surveillance systems, thereby eliciting defense responses. The effectors that elicit plant immune response can be recognized by resistance proteins, including intracellular nucleotide binding leucine-rich repeat receptors (NLRs) and the receptor-like proteins (RLPs) containing extracellular leucine-rich repeat^46,47^. Interestingly, our results found that *C*. *albicans-* derived Sel1 can be surveilled by TLR2/TLR4, which also contain extracellular leucine-rich repeats for ligand recognition, thus supporting the notion that there exist evolutionarily conserved pathogenic traits between plant and human fungal pathogens.

While it is a common theme that invaded pathogens can be recognized by multiple innate receptors simultaneously, *C*. *albicans* seems to be exceptional in its ability to engage large number of innate receptors across at least three disciplines like TLRs, CLRs, and NLRs. Not surprisingly, *C*. *albicans* has evolved a large repertoire of PAMPs. It was well accepted that the polysaccharide structures on the cell wall represent the main PAMPs of *C*. *albicans* recognized by host PRRs, with dectin-1 being the well-studied receptor for β-glucans^48,49^ and dectin-2/3, DC-SIGN, MR, MINCLE recognizing mannans and mannoproteins^50–53^. However, increased evidences have suggested that substances beyond the cell wall may serve as PAMPs activating host immune response as well, including Candidalysin, SAPs, and Pra1. Like Candidalysin, Sel1 is also capable of inducing cytokines and chemokines in the host, thus contributing to the overall magnitude of proinflammatory response mounted against invading *C*. *albicans*. However, unlike Candidalysin, Sel1 expression does not rely on the hyphae transition per se, but depending on conditions like nitrogen limitation and serum instead (Fig. 1e–h). Besides, while Candidalysin can trigger epithelium lysis, Sel1 likely primarily acts on innate immune cells such as macrophages and DCs, promoting inflammation without directly affecting cell survival (Supplementary Figure 5h). Hence, it is possible that Candidalysin and Sel1 act in temporally and spatially distinct manners during fungal infection, with Candidalysin responsible for breaching the mucosae epithelial layer at the early stage^54^, and Sel1 being recognized by immune system to provoke host defense during dissemination. Interestingly, another *Candida*-secreted protein Pra1 has been shown to be involved in the recruitment of leukocytes during systemic infection, protecting host against *C*. *albicans* infection^27,28^, raising the possibility that Sel1 may work together with Pra1 to promote immune response during *Candida* infection. In support of this hypothesis, ablation of *PRA1* or *SEL1* in *C*. *albicans* led to the same phenotypic manifestation, including reduced inflammatory response and augmented pathogenesis during systemic candidiasis (Fig. 5)^28^. Collectively, studies from others and us suggest that *C*. *albicans* can deploy a diversity of secreted proteins to induce inflammatory responses, and they work in concert to strike a delicate balance between fungal propagation and host survival.

While TLR2 and TLR4 have been widely associated with the recognition of PAMPs containing glycolipids, such as bacterial lipoprotein and LPS, as well as fungal phospholipomannan and *O*-linked mannan, our study revealed an interesting scenario that PAMPs of protein nature could also be scrutinized by TLR2 and TLR4. It is well known that TLR2 can form heterodimers with TLR1 or TLR6 to recognize Gram-positive bacteria-derived lipoprotein and peptidoglycan^55–58^, or part with dectin-1 to recognize fungal zymosan^15^. On the other hand, the recognition and immune activation of LPS by TLR4 requires co-receptors MD2^59,60^ and CD14^61,62^. The collaboration between TLR2 and TLR4 in sensing Sel1 proteins in this study provides yet another paradigm that two innate receptors cooperate in the recognition of the same PAMP.

In summary, we identified Sel1 as a novel secreted cysteine-rich protein in opportunistic human pathogen *C*. *albicans*. Our results indicated that Sel1 is largely induced and secreted under nitrogen-limitation and serum-abundant conditions, like the blood environment, thus rendering it a potential diagnostic marker for *C*. *albicans* infection. We also demonstrated Sel1 ability to promote inflammatory response through the concomitant engagement of TLR2 and TLR4 in macrophages and DCs. Considering increasingly prevalent fungal infection, Sel1 might be harnessed for the treatment of *C*. *albicans* infection based on its immune-activating ability in the future.

## Methods

### Mice

The ICR and C57BL/6 wild-type mice were purchased from Shanghai Laboratory Animal Center (SLAC). The immune-deficient mice (*Myd88*^−/−^, *Trif*^−/−^, *Tlr2*^−/−^, *Tlr4*^−/−^, *Tlr5*^−/−^, *Tlr7*^−/−^, *Il1r*^−/−^) were obtained from Jackson Labs. *Shp*2 floxed:LysM-cre mice were generated by breeding Shp2 floxed mice into LysM-cre strain^5^. The *Tlr2*^−/−^*Tlr4*^−/−^ and *Myd88*^−/−^*Trif*^−/−^ mice were generated in this study. The experiments were conducted in individual ventilated cages in a pathogen-free facility at Shanghai Institute of Biochemistry and Cell Biology following a protocol approved by the Institutional Animal Care and Use Committee of the Shanghai Institutes for Biological Sciences, Chinese Academy of Sciences.

### BMDMs, BMDCs, PBMCs, and cell lines

BMDMs were prepared by methods described previously^5^. Briefly, bone marrow cells were obtained by flushing femurs and tibia of C57BL/6 mice (6–8-weeks old) with BMDM culture medium (RPMI-1640 medium containing 10% FBS, 30% L929 conditioned medium, and 1% penicillin–streptomycin). On day 4, nonadherent cells were removed and fresh BMDM culture medium was added. On day 5, BMDMs were seeded into 6-well plate at 1.5 × 10^6^ cells/well, and used on days 7–9.

BMDCs were prepared by methods described previously^5^. Briefly, bone marrow cells were isolated by flushing of the femurs and tibia of 6- to 8-week-old C57BL/6 mice with RPMI-1640 medium (Invitrogen). Red blood cells were lysed with ACK lysis buffer (0.15 M NH_4_Cl, 1 mM KHClO_3_, and 0.1 mM Na_2_EDTA, pH 7.3). Then, cells were cultured in BMDC culture medium (RPMI-1640 medium containing 10% FBS, 10 ng/ml IL-4, 5% conditioned medium, 2 mM l-glutamine, and 200 μM β-mercaptoethanol). The BMDC culture medium was replaced every 2 days. On day 9, nonadherent cells were collected by centrifugation and then resuspended in BMDC culture medium for use.

PBMCs were isolated from blood samples obtained from healthy volunteers by density gradient centrifugation using Ficoll-Paque PLUS (GE Healthcare), and the mononuclear cell layer at the interface was taken. The PBMCs was then washed twice with phosphate-buffered saline (PBS; pH 7.4) and resuspended in RPMI-1640 medium (supplemented with 2 mM l-glutamine, 1 mM pyruvate, 10% FBS) for use. This study was approved by the Institutional Review Board and Human Ethics Committee of Affiliated Cancer Hospital and Institute of Guangzhou Medical University, and the informed consents were obtained from all the subjects.

Raw 264.7 cells (ATCC® TIB-71) were cultured in DMEM containing 10% FBS and 1% penicillin–streptomycin. Raw 264.7 cells were plated at 0.8 × 10^6^ cells/well in 6-well plate 24 h before use. L929 cells (ATCC® CRL-6364) (1.0 × 10^6^ cells) were cultured in 100 ml RPMI-1640 medium containing 10% FBS and 1% penicillin–streptomycin for 7–8 days, and then the culture supernatant was collected and stored at −80 °C for use. J558L cells (ATCC® TIB-6) were cultured in Iscove's modified Dulbecco's medium (IMDM; 20% FBS) without G418 until the cell confluence reached 90%, and then in IMDM (5% FBS) with G418 (1 mg/ml) until cell confluence became about 90%, followed by incubating in 175 flasks in IMDM (5% FBS) without G418 for 3–5 days, and the conditioned media were collected. All the cells were incubated at 37 °C with 5% CO_2_.

### *C. albicans* strains

*C*. *albicans* wild-type strain SC5314 was used for *SEL1* induction experiments. *C*. *albicans sel1/sel1* deleting mutant strain was generated by PCR-based homologous recombination^63^. Briefly, two disrupting vectors pCPC48/49 carrying *CmLEU2*, *CdHIS1* markers were used as templates, and three sets of primers (*SEL1*-39s/*SEL1*+577a, *SEL1*-69s/*SEL1*+590a, *SEL1*-100s/*SEL1*+621a) were used to amplify 5′ and 3′ fragments of *SEL1*, and then assembled with LoxP-CmLEU2-LoxP (PLP) or LoxP-CdHIS1-LoxP (PHP) respectively by fusion PCR method. The two *SEL1* deleting cassettes PLP and PHP were introduced into SN152 strain subsequently to generate the *sel1/sel1* mutant. The wild-type strain SN250 was used for Sel1 functional comparison analysis. The Sel1-HA expression strain was constructed as previously described^64^. Two sets of primers (*SEL1*-F1-3HA/*SEL1*-R1-3HA, *SEL1*-F2-3HA/*SEL1*-R2-3HA) were used to amplify pCPC61 (carrying 3HA/*CmLEU2* markers), and the amplified target cassette (*SEL1* homologous sequence-3HA/CmLEU2) was introduced into SN152 to generate a strain harboring C-terminal HA-tagged Sel1 from endogenous *SEL1* promoter. The strains used in this study are described in Supplementary Table 2.

### Media, growth conditions, and *SEL1* induction

*C*. *albicans* strains were routinely grown on YPD medium (1% yeast extract, 2% peptone, 2% dextrose) or on synthetic complete medium SCD (0.17% Difco yeast nitrogen base w/o ammonium sulfate, 0.5% (37 mM) ammonium sulfate_,_ auxotrophic supplements and 2% dextrose) for selection of prototropic strains. The other media used for cells culture are synthetic low dextrose medium (SCLD; 0.1% dextrose), synthetic medium without animo acids (SCD-aa), synthetic low ammonium medium (SCDLA; 50 μM ammonium sulfate), SC with 2% *N*-acetyglucosamine (SC+N), SC with 1% lactate (SC+lac), and SC with 2% glycerol (SC+gly). Lee’s medium (5 g (NH_4_)_2_SO_4_, 2.5 g K_2_HPO_4_ Anhydrous, 5 g CaCl_2_, 0.5 g alanine, 1.3 g leucine, 1 g lysine, 0.1 g methionine, 0.072 g ornithine, 0.5 g proline, 0.5 g threonine, 0.5 g Phenylalanine, 0.085 g Arginine, 10 ml 20 mg/ml MgSO_4_, 1 ml 2 mg/ml biotin, 0.2 mM ZnSO_4_, 1 mM FeCl_3_, 1 M MgCl_2_•6H_2_O, 0.25 mM CuSO_4_•5H_2_O with 2% glucose, pH 7.0, in 1 L). Lee’s low ammonium medium (50 μM ammonium sulfate). *C*. *albicans* wild-type strain SC5314 was grown overnight in liquid YPD at 25 °C, pelleted, washed twice with PBS, and diluted 1:100 into different fresh media at 25 °C for 6 h to detect *SEL1* expression, unless otherwise indicated. Then, the fungal cells were collected for RNA extraction and qRT-PCR analysis. The relative *SEL1* expression level was normalized by the *SEL1* level from overnight culture in YPD.

### Growth rate and yeast-to-hyphae transition

For cell growth rate analysis, WT or *sel1*/*sel1* overnight cultures *C*. *albicans* were washed and resuspended in the same volume of PBS, inoculated into fresh liquid YPD, and grown at 25 °C, and then OD_600_ values were measured at the indicated time points. For yeast-to-hyphae transition, the washed overnight yeast cells were induced in YPD medium containing 10% serum at 37 °C. For hyphae-to-yeast reversion, the induced hyphae cells were transferred into fresh YPD medium and cultured at 25 °C. Cells were taken for photograph at the indicated time points. For embedded filamentous growth, overnight *C*. *albicans* culture were mixed with YPS agar (1% yeast extract, 2% peptone, 1% sucrose, and 1% agar) and incubated at 37 °C for 4 days. For morphology determination during BMDM infection, BMDMs were grown on coverslips at 5 × 10^5^ cells/well in 12-well plate, then infected with wild-type or *sel1*/*sel1* mutant *C*. *albicans* cells (multiplicity of infection (MOI) = 2), followed by microscopy at indicated time.

### RNA preparation and qRT-PCR analysis

RNA extraction of *C*. *albicans* cells was performed as described by Lane et al.^65^. RNA of mammalian cells or tissues was extracted with TRIZOL (Invitrogen) according to the manufacturer’s instruction. Complementary DNAs (cDNAs) were reversely transcribed from 1 μg of total RNAs by TIANscript RT Kit (KR104). Real-time PCRs were carried out with SuperReal PreMix Plus (SYBR Green, FP205) on ABI 7900HT Fast Real-time PCR System. Relative expression levels of target genes were quantitatively normalized against the expression of *ACT* using _ΔΔ_CT method. The cDNAs were also used for PCR, and the PCR products were detected on 2% agarose gel. All the PCR primers used in this study are described in Supplementary Table 1.

### Plasmid construction

For Sel1 expression in *E. coli*, full-length *SEL1* gene was amplified from the *C*. *albicans* genomic DNA and inserted into the pET-28a with an N-terminal 6 × His tag (Novagen), generating pET-28a-Sel1. The pET-28a-Sel1CS mutant (7 cysteines to 7 serines) was constructed based on overlapping PCR approach from the pET-28a-Sel1. The primers used for construction are in Supplementary Table 1. For Sel1 expression in mammalian cells, full-length *SEL1* gene was synthesized with mammalian preferred codons, and the Sel1 coding region lacking signal peptide was inserted into the pcDNA3.0 with an N-terminal Flag tag, generating pcDNA3.0-Flag-Sel1.

### Protein purification

The expression plasmids (His-Sel1, His-Sel1 CS mutant) were transformed and the recombinant proteins were expressed in *E*. *coli* BL21 (DE3) Codon-Plus strain (Novagen). The cells were grown at 37 °C in LB medium containing 0.05 mg/ml kanamycin to log phase and induced with 0.1 mM isopropyl β-d-1-thiogalactopyranoside (IPTG) at 16 °C for 24 h. The cells were collected and sonicated in a lysis buffer (50 mM Tris pH 8.0, 300 mM NaCl, 10 mM imidazole, 0.1% Triton X-100, 1 mM PMSF). The lysate was followed by centrifugation to get rid of the cell debris, and target proteins were purified by affinity chromatography using a Ni-NTA column (Qiagen) and then gel filtration chromatography using a Superdex 200 16/60 (preparative grade) column (0.4 ml/min, GE Healthcare) and HiTrap Q HP column (1 ml/min, GE Healthcare). The purified protein was then subjected to LPS elimination by endotoxin removal kit (Pierce High Capacity Endotoxin Removal Spin Column). The purified protein was filtered with 0.22 μm filter, and LPS level was determined by LAL method (Pierce LAL Chromogenic Endotoxin Quantitation Kit). The protein was stored at −80 °C for further use.

### Western blotting and Coomassie blue staining

The whole cell lysate of *C*. *albicans* cells was made in lysis buffer supplemented with protease inhibitor complete mini and PhosSTOP phosphatase inhibitor. To obtain whole cell lysates of mammalian cells, cells were lysed in lysis buffer for 30 min on ice, and cell debris was cleared by centrifugation at 17,000 × *g* for 15 min. The protein concentration of the cell lysate was measured by Bradford method, and the cell lysate was mixed with SDS-loading buffer and boiled at 100 °C for 10 min. For secreted Sel1 detection, culture supernatant was mixed with SDS-loading buffer and boiled at 100 °C for 10 min. The samples were fractionated by sodium dodecyl sulfate–polyacrylamide gel electrophoresis (SDS-PAGE) gel, transferred to NC membrane, and then probed with the appropriate antibodies. The uncropped and unprocessed scans of the most important blots which were used for Figures and Supplementary Figures have been supplied as Source Data file in the Supplementary Information. *E*. *coli* purified proteins were fractionated by SDS-PAGE gel and incubated in the Coomassie blue staining buffer (Coomassie brilliant blue 2.5 g, 45% carbinol, 10% acetic acid in 1 L ddH_2_O) at room temperature for 1 h. Then, the gel was washed in the washing buffer until the gel background was clean (25% carbinol, 8% acetic acid).

### Immunoprecipitation and pull-down assay

The plasmids expressing TLR2, TLR4-HA, and Flag-Sel1 were transfected into HEK293T cells, respectively. The transfected cells were lysed in a lysis buffer (50 mM Tris, pH 7.5, 150 mM NaCl, 1% Triton X-100, 1 mM EDTA, 1 mM NaF, 1 mM Na_3_VO_4_) supplemented with protease inhibitor cocktail (cOmplete Mini EDTA-free, Roche) and PhosSTOP, incubated in ice for 40 min, and then cell debris were cleared by centrifugation at 17,000 × *g* for 15 min. The Sel1-flag protein containing lysates were immunoprecipitated by incubation overnight with ANTI-FLAG M2 Affinity Agarose. Then, the blank anti-flag agarose or the Sel1-flag bound agarose were incubated with cell lysates containing TLR2 or TLR4-HA respectively at 4 °C for 3 h. The immunoprecipitates were then washed three times with buffer (50 mM Tris, pH 8.0, 300 mM NaCl, 1% Triton X-100, 1 mM EDTA, and 0.1% SDS). Proteins were eluted with 2× SDS-loading buffer. After being boiled for 10 min, the samples were fractionated by 8% SDS-PAGE and 12% SDS-PAGE for the following western blotting detection.

### Cell stimulation

For stimulation by *E*. *coli* purified proteins, the *E*. *coli* purified Sel1 and AldoB were used for macrophage stimulation. Pretreated Sel1 was prepared as follows: 300 ng of Sel1 was incubated with 20 μg DNase, 20 μg RNase, 100 μg PMB, or 40 μg proteinase K, respectively, in 20 μl PBS for 15 min, and then the mixtures were used for BMDM stimulation. BMDMs are also stimulated with different stimuli as follows: DNase (20 μg/ml), RNase (20 μg/ml), PMB (100 μg/ml), proteinase K (40 μg/ml), P3C (Pam3CSK4, 100 ng/ml), LPS (100 ng/ml), pI:C (polyinosinic:polycytidylic acid, 10 μg/ml), and Zymd (100 μg/ml). The 3 h stimulated BMDMs were used for detection of cytokine production, and 30 min stimulated BMDMs were used for analysis of signaling activation. For stimulation by *C*. *albicans* secreted proteins, overnight culture of wild-type *C*. *albicans* SN250 or *sel1/sel1* mutant cells were pelleted, washed, and inoculated into RPMI-1640 medium with 10% serum, and further cultured at 25 °C for 12 h. The culture media were collected and filtrated with 0.22 μm filter and used for stimulating BMDMs. After 3 h of stimulation with the supernatants, BMDMs were harvested for cytokine analysis.

### Macrophage infection with *C*. *albicans*

For expression study on candidate SCPs, raw 264.7 cells or BMDMs were infected with wild-type *C*. *albicans* strain SC5314 (MOI = 2), and then fungal cells were collected at various time points, and further used for RNA extraction and qRT-PCR analysis. For phagocytosis and killing of *C*. *albicans* cells, BMDMs were plated at 7.5 × 10^5^ cells/well in 12-well plate, and infected with *C*. *albicans* wild-type SN250 or *sel1*/*sel1* mutant cells (MOI = 0.5). At 0.5 h post infection, after washing away free fungal cells with PBS, the BMDMs were scraped, lysed in a lysis buffer (50 mM Tris, pH 7.5, 150 mM NaCl, 1% Triton X-100, 1 mM EDTA), resuspended, serially diluted, and plated onto YPD agar. For longer time phagocytosis, BMDMs were scraped at 6 h or 12 h post infection, and the phagocytized fungal cells were plated onto YPD agar. Fungal colony-forming units were counted after incubation at 30 °C for 48 h.

### Mouse infection with *C*. *albicans*

Live *C*. *albicans* cells were injected intravenously into ICR mice (male, 18–21 g), C57BL/6 mice (male, 6 weeks old), or *Tlr2*^−/−^*Tlr4*^−/−^ mice (male, 6 weeks old). Infected mice were monitored daily for weight loss and survival. On day 5 post infection, livers, spleens and kidneys of ICR mice were dissected, and the tissue homogenates were serially diluted and plated onto YPD agar. Fungal colony-forming units were counted after incubation at 30 °C for 48 h. The fungal burden was assessed by counting CFU. Kidneys are also homogenized in TRIZOL to extract RNA for cytokines production analysis.

### ELISA analysis

The supernatants of homogenized kidneys were harvested and the amounts of TNF and IL-6 were measured with ELISA kits according to the manufacturers’ recommendations (eBioscience and R&D Systems). The whole blood was taken from mice hearts on day 5 post infection, the serum was harvested, and the amount of TNF and IL-6 was measured in the same way.

### Quantification and statistical analysis

The log-rank test (Mantel–Cox) and Student’s *t*-test were used to analyze the significance of statistics, and the *P* value of <0.05 was considered significant.

### Reporting summary

Further information on experimental design is available in the Nature Research Reporting Summary linked to this article.

## Supplementary information

Supplementary Information

Reporting Summary

## Data Availability

The authors declare that the main data supporting the findings of this study are available within the article and its Supplementary Information. Extra data that support the findings of this study are available from the corresponding authors upon reasonable request.
