# Supplementary material for: A small secreted protein triggers a TLR2/4-dependent inflammatory response during invasive Candida albicans infection
Source: Nat Commun. 2019 Mar 4;10:1015. doi: 10.1038/s41467-019-08950-3 (PMC6399272; doi:10.1038/s41467-019-08950-3)

Supplementary information

A small secreted protein triggers a TLR2/4-dependent inflammatory response during invasive *Candida albicans* infection

Wang et al.

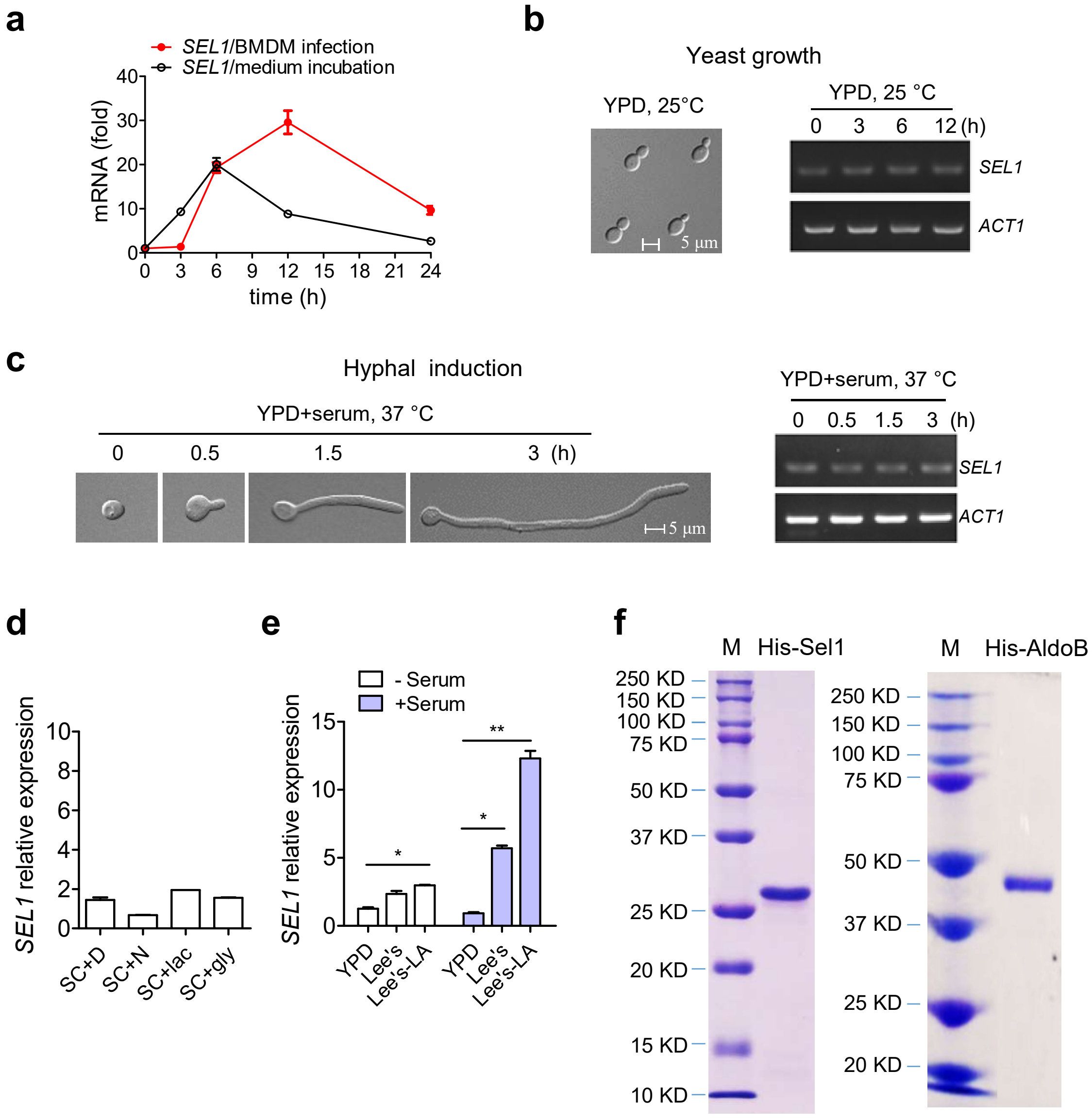

Supplementary Figure 1. The expression of endogenous and recombinant Sel1. (a) *C. albicans* SC5314 (MOI=2) were incubated with BMDMs or cultured in RPMI 1640 medium containing 10% FBS for indicated times, then fungal cells were collected for *SEL1* expression analysis by qRT-PCR. **(b, c)** *C. albicans* SC5314 strain was cultured in yeast growth condition **(b)** or hyphae induction condition **(c)**, and cells were collected for morphology determination (left) by microscopy and *SEL1* expression analysis (right) by RT-PCR at different times. **(d, e)** SC5314 were cultured in different media (at 25°C for 6 h) for *SEL1* expression analysis by qRT-PCR. Different carbon source of Synthetic Complete medium **(d)** and Lee's modified media **(e)** were used. D, dextrose. N, N-acety-glucosamine. Lac, lactate. Gly, glycerol. LA, low ammonia**.** Data were relative to the un-induced overnight culture. **(f)** The purified His-Sel1 or His-AldoB proteins were resolved by SDS-PAGE and stained by Coomassie brilliant blue. Data are representative of at least three **(a)** or two **(b-e)** independent experiments with similar results. Bars, mean±SD. Fold induction of RNA levels represents the relative levels of induced RNAs to the un-induced RNAs in *C. albicans* cells **(a-e)**. *p < 0.05, **p < 0.01, ***p < 0.001, by Student's *t*-test.

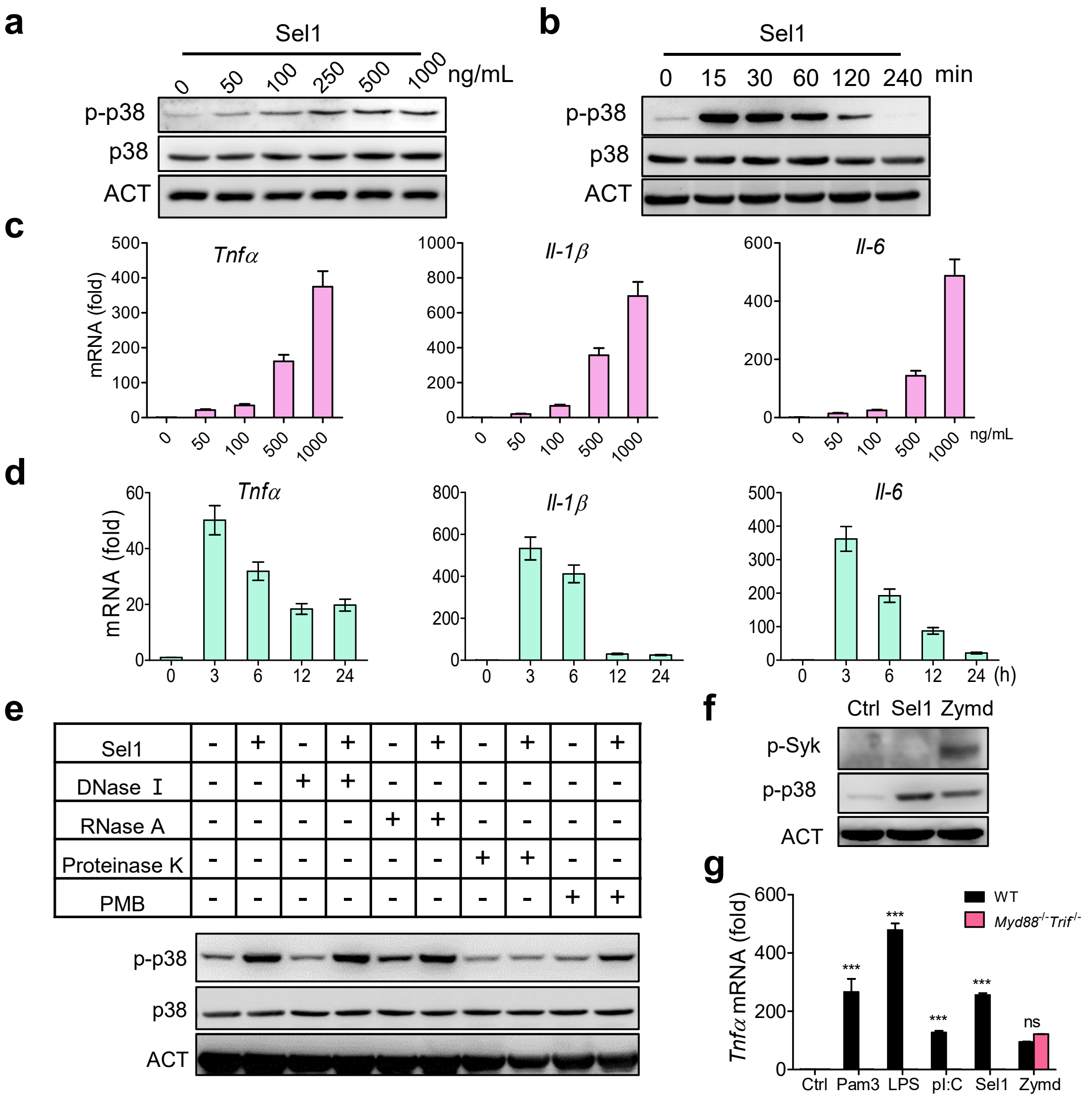

Supplementary Figure 2. Sel1 does not activate CLR-dependent Syk phosphorylation. (**a-b**) BMDMs were stimulated with Sel1 in different doses for 0.5 h **(a)** or with 300 ng/ml of Sel1 for different time points **(b),** and the signaling pathways were examined by Western blotting. **(c-d)** BMDCs were stimulated with different doses of Sel1 for 3 h **(c)** or with 300 ng/ml of Sel1 for different time points **(d)**, and the cytokines production was assessed by qRT-PCR. **(e)** BMDMs were stimulated with Sel1 pretreated with proteinase K, DNase, RNase or PMB respectively, and the signaling events were analyzed by Western blotting. **(f)** BMDMs primed with IL-4 (10 ng/ml, 12 h) were stimulated with Sel1 or Zymd, the signaling activation was examined by Western blotting. Zymd, the glucan zymosan depleted of TLR ligands. **(g)** WT and *Myd88*$^{-/-}$*Trif*$^{-/-}$ BMDMs were stimulated with various TLR/CLR ligands, and the induction of *Tnfα* was assessed by qRT-PCR. Data are representative of at least three independent experiments with similar results. Data represent the average expression levels of genes from at least two independent experiments **(c-d, g)**. Bars, mean ± SEM. $*p < 0.05$, $**p < 0.01$, $***p < 0.001$, by Student's *t*-test.

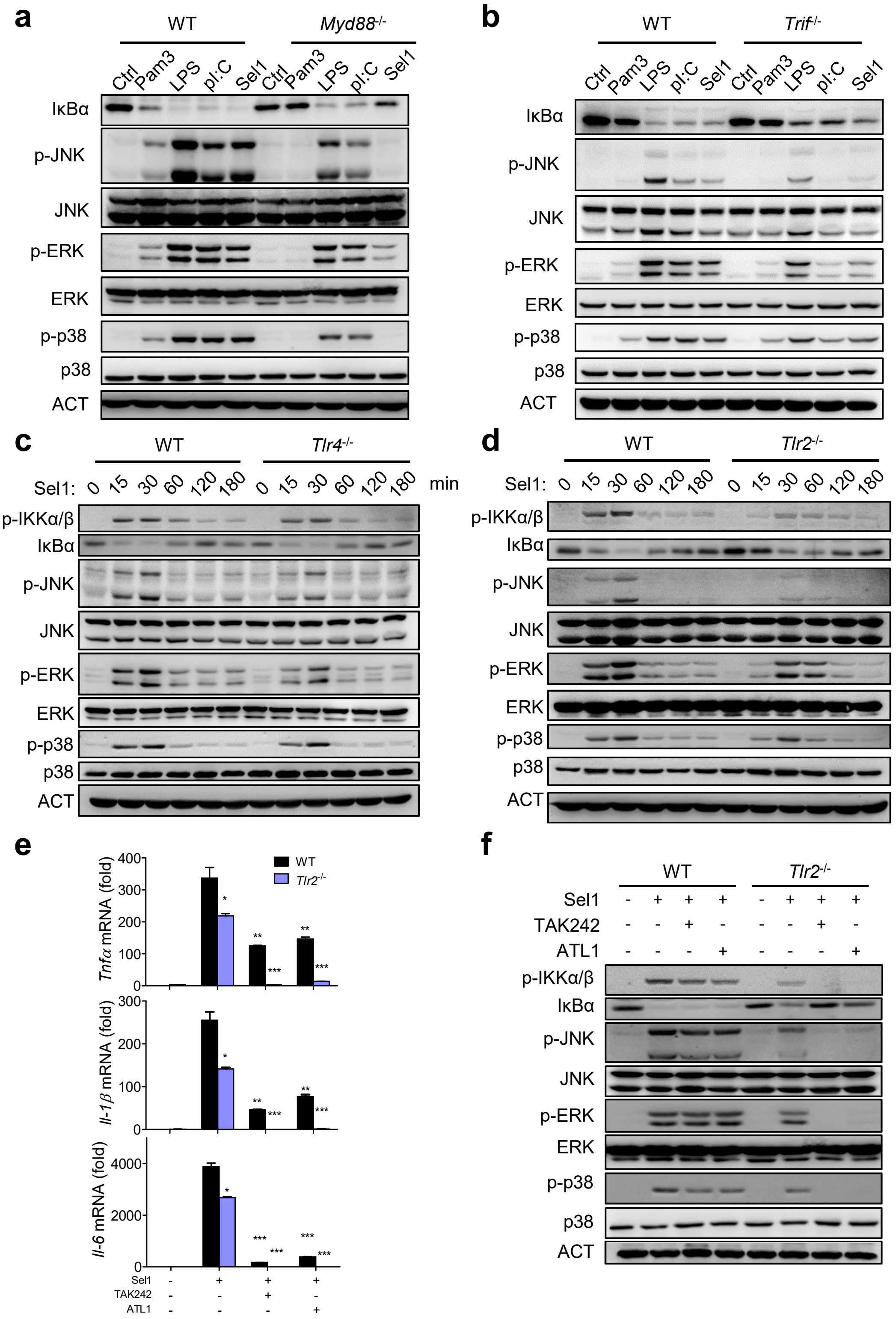

Supplementary Figure 3. Sel1 activates TLR2/4-dependent signaling events in BMDMs. (a, b) *Myd88*$^{-/-}$ BMDMs **(a)** and *Trif*$^{-/-}$ BMDMs **(b)** were stimulated with various TLR ligands or Sel1, and the NF-κB and MAPK signaling pathways were examined by respective antibodies through Western blotting.

Supplementary Figure 3. Sel1 activates TLR2/4-dependent signaling events in BMDMs. (c, d) *Tlr4*$^{-/-}$ BMDMs **(c)** and *Tlr2*$^{-/-}$ BMDMs **(d)** were stimulated with Sel1 for different time points, and the NF-κB and MAPK signaling pathways were examined by Western blotting. **(e, f)** *Tlr2*$^{-/-}$ BMDMs were untreated or treated with TLR4 inhibitor TAK242 or TLR4 antagonist atractylenolide I (ATL1). The cytokine production was assessed by qRT-PCR (**e**)**,** and signaling pathways were examined by Western blotting (**f**)**.** Data are representative of at least three independent experiments with similar results. Data represent the average expression levels of each gene compared to the Ctrl WT cells from at least two independent experiments (**e**). Bars, mean ± SEM. *p < 0.05, **p < 0.01, ***p < 0.001, by Student's *t*-test.

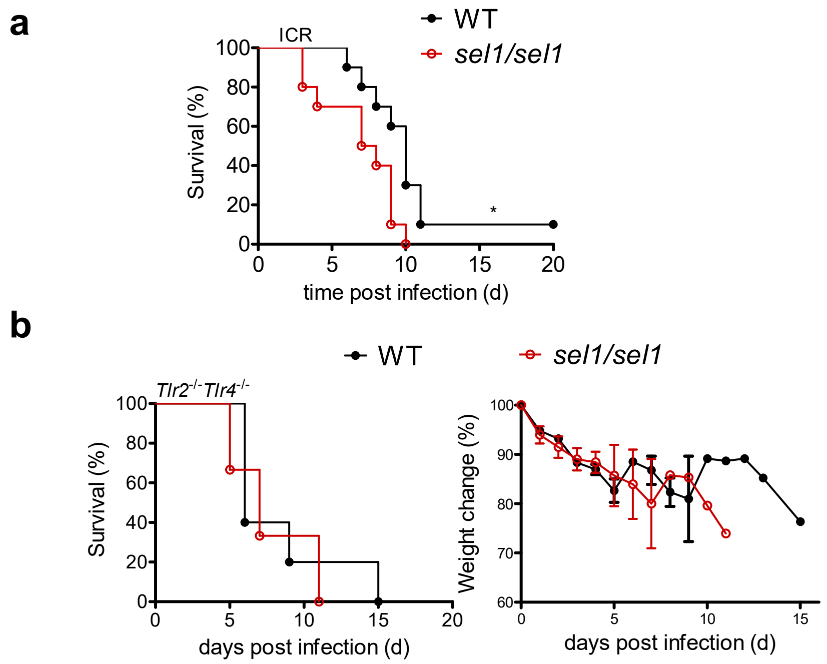

Supplementary Figure 4. Systemic mice infection assay. (a) Percent survival of ICR wild type mice infected intravenously (male, 18-21 g, 5×10^5 CA cells/mouse, n=10). (b) *Tlr2*$^{-/-}$*Tlr4*$^{-/-}$ mice (male, 6 w, n=4, 5×10^5 CA cells/mouse) were infected with WT or *sel1/sel1 C. albicans*, and the percent survival (left) and weight change (right) were shown.

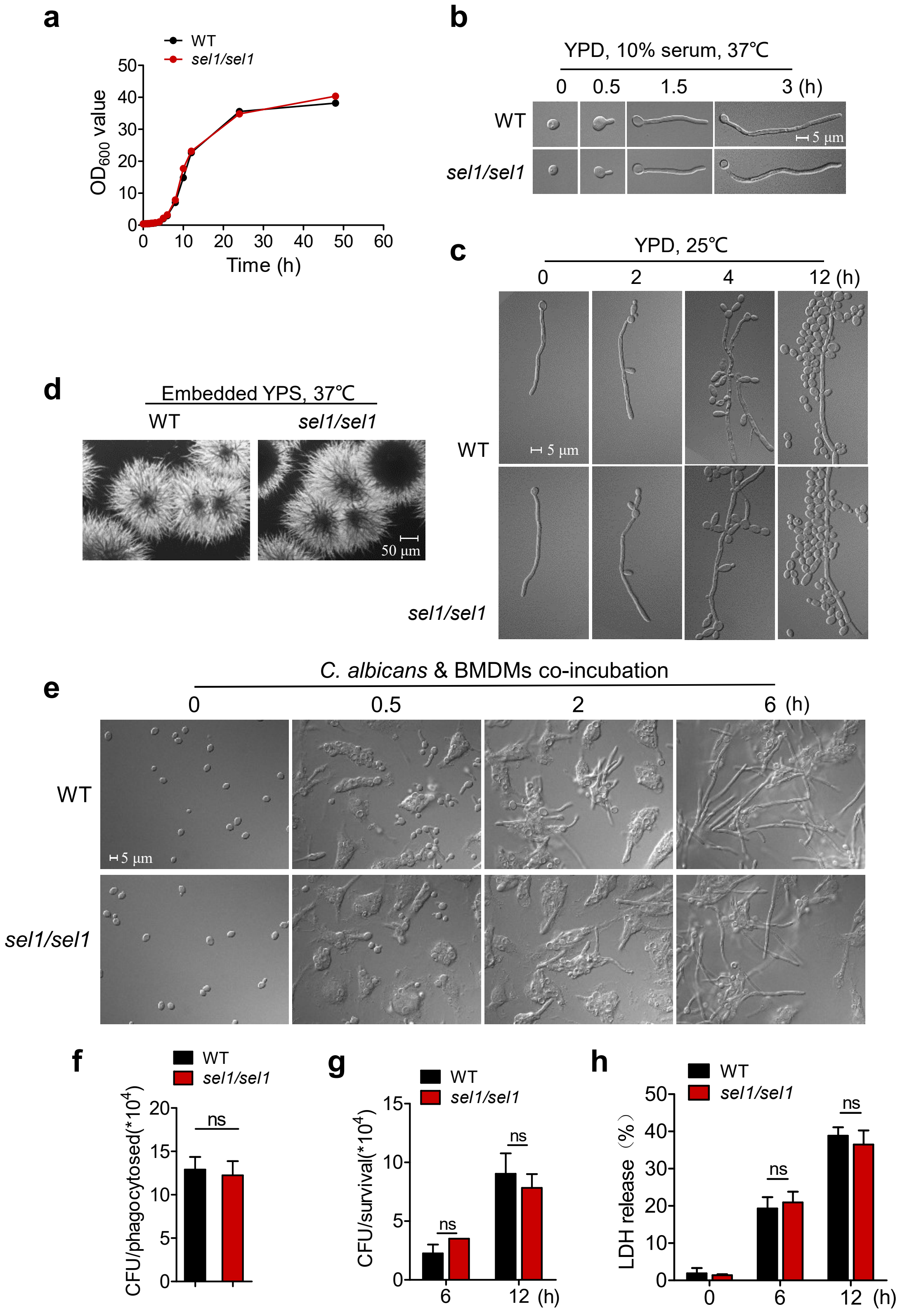

a
WT
sel1/sel1
OD600 value
Time (h)
b
YPD, 10% serum, 37℃
0 0.5 1.5 3 (h)
WT
sel1/sel1
5 μm
c
YPD, 25℃
0 2 4 12 (h)
WT
sel1/sel1
5 μm
d
Embedded YPS, 37℃
WT
sel1/sel1
50 μm
e
C. albicans & BMDMs co-incubation
0 0.5 2 6 (h)
WT
sel1/sel1
5 μm
f
WT
sel1/sel1
CFU/phagocytosed(*10^4)
ns
g
WT
sel1/sel1
CFU/survival(*10^4)
ns
6 12 (h)
h
WT
sel1/sel1
LDH release（%）
ns
0 6 12 (h)

Supplementary Figure 5. *SEL1* is not involved in the growth, morphology or macrophage phagocytosis of *C. albicans*.(a) WT and *sel1/sel1 C. albicans* cells were cultured in YPD medium at 25°C for 48 h, and the OD_{600} value was measured at indicated time points. **(b, c)** WT and sel1/sel1 *C. albicans* yeasts were cultured in hyphae induction condition **(b)** or hyphae cultured in yeast growth condition **(c)**, then cells were photographed at different time points by microscopy. **(d)** WT and *sel1/sel1 C. albicans* were embedded in YPS solid medium for 4 days, and then colonies were taken for observation by microscopy. **(e)** BMDMs were grown on coverslips at 5×10^5 cells/well in 12-well plate, then infected by WT or *sel1/sel1 C. albicans* cells (MOI=2). At the indicated time points, cells were fixed in 4 % PFA and observed by microscopy. **(f-h)** BMDMs were plated at 5×10^5 cells/well in 12-well plate, followed by infection with WT or *sel1/sel1 C. albicans* (MOI=0.5). The phagocytized fungal cells at 0.5 h post infection **(f)** or the survived fungal cells at 6 h/12 h post infection **(g)** were collected, diluted and plated on YPD agar. The results are presented as Colony-Forming Units. **(h)** BMDMs were plated at 2.5×10^5 cells/well in 24-well plate, followed by infection with WT or *sel1/sel1 C. albicans* cells (MOI=0.5) for indicated times. The LDH content was determined using CytoTox-ONE™ Homogeneous Membrane Integrity Assay (Promega).

Supplementary Table 1

Purpose and features	Primers	Sequence
For flag-Sel1 expression in mammalian cells	Sel1-pCDNA3.0-F	TCCGGAATTCTGCAGTTCAACCTGCTCCACGATGA
	Sel1-pCDNA3.0-R	GGCCGCCAGTGTGATCTATTTGGCACAGGCGCC
	pCDNA3.0-F	ATCACACTGGCGGCCGCTC
	pCDNA3.0-R	CTGCAGAATTCCGGATCCG
For His-Sel1 expression in *E.coli*	pET-28a-F	CGTCGACAAGCTTGCGGCCG
	pET-28a-R	TTGCTGTCCACCAGTCATGCTAGC
	SEL1-pET28a-F	ACTGGTGGACAGCAAATGTTCAATTTATTACATGATGATA AAGGTTTT
	SEL1-pET28a-R	GCAAGCTTGTCGACGTTTAGCACAAGCACCACCAAC
For *sel1/sel1* mutant construction	SEL1-39s	CAAATAAGCTTGAATTACTAGAATCATCAAAGTAACAAAT GCCACCTGACGTCTAAGAA
	SEL1+577a	AAAAAAACCGTTAATTCCTTTTTTTTAAAGTTTCTCTTTGG CCTTTTGCTCACATGTTC
	SEL1-69s	ATTTCTTTATCCTTTTGTTTTTTTGATAAACAAATAAGCTT GAATTACTAGAATCATCA
	SEL1+590a	TCACTTATTCAATCAATAGAGGAAACTAAATAAAAAAAAA CCGTTAATTCCTTTTTTTT
	SEL1-100s	CTTCATAAATATATATATATATATAATTTAGTAATTTCTTTATC CTTTTGTTTTTTTGATA
	SEL1+621a	AATTCATTTCATATTTGTTCTTGTATATATATCACTTATTC AATCAATAGAGGAAACTA
For *C. albicans (Sel1-HA)* construction	SEL1-F1-3HA	CTACTAAAGCTGAAGTCGTTGGTGGTGCTTGTGCTAAAA GCGCGCCAATTAGATCCGTG
	SEL1-R1-3HA	AAAACCGTTAATTCCTTTTTTTTAAAGTTTCTCTTTTTATT CTTTCCTGCGTTATCCTG
	SEL1-F2-3HA	GTTCCAGCTCAAGTCAGATGTTACCATAAATTAGAACAA GCTACTAAAGCTGAAGTCGT
	SEL1-R2-3HA	ATATCACTTATTCAATCAATAGAGGAAACTAAATAAAAAA AAACCGTTAATTCCTTTTT
For His-Sel1 C/S mutant expression in E.coli	SEL1-CSmut1-F	CATACTCATGAAACTAAAAGUACTGGTAAATATGCTAAAG
	SEL1-CSmut1-R	CTTTAGCATATTTACCAGTACTTTTAGTTTCATGAGTATG
	SEL1-CSmut2-F	GATAGTTCTTTAAAACAAAGUTTGAACAAAGAATTTG
	SEL1-CSmut2-R	CAAATTCTTTGTTCAAACTTTGTTTTAAAGAACTATC
	SEL1-CSmut3-F	GCTGAAACTAGTGAAAGUGGATTTGAATTTATTG
	SEL1-CSmut3-R	CAATAAATTCAAATCCACTTTCACTAGTTTCAGC
	SEL1-CSmut4-F	GAATATAATTCTGCTTCTGGTAGUTCTAGTGATGCTAC
	SEL1-CSmut4-R	GTAGCATCACTAGAACTACCAGAAGCAGAATTATATTC
	SEL1-CSmut5-F	GCTACTAAATATTCAAGUCTTTTGGATGTTATG

	SEL1-CSmut5-R	CATAACATCCAAAAGACTTGAATATTTAGTAGC
	SEL1-CSmut6-F	CCAGCTCAAGTCAGAAGUTACCATAAATTAGAAC
	SEL1-CSmut6-R	GTTCTAATTTATGGTAACTTCTGACTTGAGCTGG
	SEL1-CSmut7-F	CATAAATTAGAACAAGCTACTAAAGCTGAAGTCGTTGGT GGTGCTAGUGCTAAA
	SEL1-CSmut7-R	TTTAGCACTAGCACCACCAACGACTTCAGCTTTAGTAGC TTGTTCTAATTTATG
For qRT-PCR detection of the indicated genes	SEL1-400s	GCTTCTGGTTGTTCTAGTGATGC
	SEL1-561a	ACCACCAACGACTTCAGCTT
	caACT-R	GCTTTTGGTGTTTGACGAGTTTCT
	caACT-F	GTGAGCCGGGAAATCTGTATAGTC
	mTNFα-F	GTCCCCAAAGGGATGAGAAGTT
	mTNFα-R	GTTTGCTACGACGTGGGCTACA
	mIL-1β-F	CAACCAACAAGTGATATTCTCCATG
	mIL-1β-R	GATCCACACTCTCCAGCTGCA
	mIL-6-F	AGATAAGCTGGAGTCACAGAAGGAG
	mIL-6-R	CGCACTAGGTTTGCCGAGTAG
	mACT-R	AGGGACAGCACAGCCTGGAT
	mACT-F	TTACCAACTGGGACGACATG

Primers used in this study

Supplementary Table 2

REAGENT or RESOURCE		SOURCE	IDENTIFIER
**Antibodies**			
p-IκBα	1:1000 used	Cell Signaling Technology	Cat#2859
p-p38	1:1000 used	Cell Signaling Technology	Cat #9211
p-JNK	1:1000 used	Cell Signaling Technology	Cat#4668
JNK	1:1000 used	Cell Signaling Technology	Cat#9252
p-IKKα/β	1:1000 used	Cell Signaling Technology	Cat#2697
p-Syk	1:1000 used	Cell Signaling Technology	Cat#2710
IκBα	1:1000 used	Santa Cruz Biotechnology	SC-1643
p38	1:1000 used	Santa Cruz Biotechnology	SC-728
p-ERK	1:1000 used	Santa Cruz Biotechnology	SC-7383
ERK	1:1000 used	Santa Cruz Biotechnology	SC-93
HA	1:1000 used	Sigma-Aldrich	H6908
H3	1:2500 used	Abcams	ab-1791
β-ACTIN	1:5000 used	Abways technology	ab2001
**Bacterial and Candida Strains**			
Trans5α Chemically Competent Cell		TRANSGEN BIOTECH	CD201-01
BL21(DE3) Chemically Competent Cell		TRANSGEN BIOTECH	CD601-01
Candida albicans SC5314 (wild type)		This paper	[1]
Candida albicans SN152 (*ura3*::imm434::*URA3*/*ura3*::imm434 *iro1*::*IRO1*/*iro1*::imm434 *his1*::*hisG*/*his1*::*hisG leu2*/*leu2 arg4*/*arg4*)		This paper	[2]
Candida albicans SN250 (*ura3*::imm434::*URA3*/*ura3*::imm434 *iro1*::*IRO1*/*iro1*::imm434 *his1*::*hisG*/*his1*::*CdHIS1 leu2*/*CmLEU2 arg4*/*arg4*)		This paper	[2]
Candida albicans sel1/*sel1* mutant (*ura3*::imm434::*URA3*/*ura3*::imm434 *iro1*::*IRO1*/*iro1*::imm434 *his1*::*hisG*/*his1*::*hisG leu2*/*leu2 arg4*/*arg4 sel1*::*PLP*/*sel1*::*PHP*)		This paper	N/A
Candida albicans SEL1-HA (*ura3*::imm434::*URA3*/*ura3*::imm434 *iro1*::*IRO1*/*iro1*::imm434 *his1*::*hisG*/*his1*::*hisG leu2*/*leu2 arg4*/*arg4 SEL1*/*sel1*::*SEL1-PLP*)		This paper	N/A
**Chemicals, Peptides, and Proteins**			
Tryptone		BD-Bacto	211705
Peptone		BD-Bacto	211677
Yeast extract BD-Bacto		BD-Bacto	212750
RPMI-1640		Hyclone	SH30809
Dulbecco's Modified Eagle Medium (DMEM)		Hyclone	SH30022
Protease inhibitor cocktail tablets (cOmplete		Roche Diagnostics	11836170001

Mini EDTA-free)		
PhosSTOP	Roche Diagnostics	4906837001
ANTI-FLAG® M2 Affinity Agarose	Sigma	A2220
Fetal bovine serum	Gibco	10099141
IL-4	R&D Systems	-
Pam3CSK4	InvivoGen	tlrl-pms
LPS	InvivoGen	-
Poly(I:C)	InvivoGen	-
Polymyxin B (PMB)	InvivoGen	tlrl-pmb
Zymd	InvivoGen	tlrl-dzn
TAK242	MCE MedChemExpress	HY-11109
Atractylenolide I (ATL1)	MCE MedChemExpress	HY-N0201
**Critical Commercial Assays**		
Pierce™ High Capacity Endotoxin Removal Spin Columns	ThermoFisher SCIENTIFIC	88274
Pierce LAL Chromogenic Endotoxin Quantitation Kit	ThermoFisher SCIENTIFIC	88282
CytoTox-ONE™ Homogeneous Membrane Integrity Assay	Promega	G7890
**Recombinant DNA**		
pET-28a(+) DNA	Novagen	69864
pET-28a-Sel1	This paper	N/A
pET-28a-Sel1(CS mutant)	This paper	N/A
pCPC48 (*LoxP-CmLEU2-LoxP* (*PLP*), *Amp*^{R} in the pUC18)	This paper	[3]
pCPC49 (*LoxP-CdHIS1-LoxP* (*PHP*), *Amp*^{R} in the pUC18)	This paper	[3]
pCPC61 (3HA-ADH1 Terminator-*LoxP-CmLEU2-LoxP* (*PLP*), *Amp*^{R} in the pUC18)	This paper	[4]
**Software**		
GraphPad Prism (version 7.0)	GraphPad Software	https://www.graphpad.com
Multi Gauge Software	Winsite	http://multi-gauge.winsite.com/

1 Fonzi, W. A. & Irwin, M. Y. Isogenic strain construction and gene mapping in Candida albicans. *Genetics* 134, 717-728 (1993).

2 Noble, S. M. & Johnson, A. D. Strains and strategies for large-scale gene deletion studies of the diploid human fungal pathogen Candida albicans. *Eukaryotic cell* 4, 298-309, doi:10.1128/EC.4.2.298-309.2005 (2005).

3 Chang, P., Fan, X. & Chen, J. Function and subcellular localization of Gcn5, a histone acetyltransferase in Candida albicans. *Fungal genetics and biology : FG & B* 81, 132-141, doi:10.1016/j.fgb.2015.01.011 (2015).

4 Chang, P., Wang, W., Igarashi, Y., Luo, F. & Chen, J. Efficient vector systems for economical and rapid epitope-tagging and overexpression in Candida albicans. *Journal of microbiological methods* 149, 14-19, doi:10.1016/j.mimet.2018.04.016 (2018).

Unprocessed scans of the most important blots

Fig. 1c

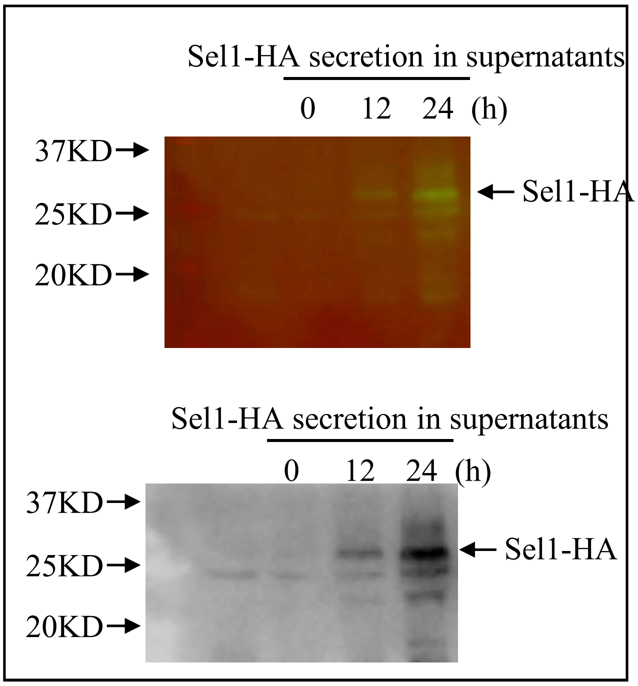

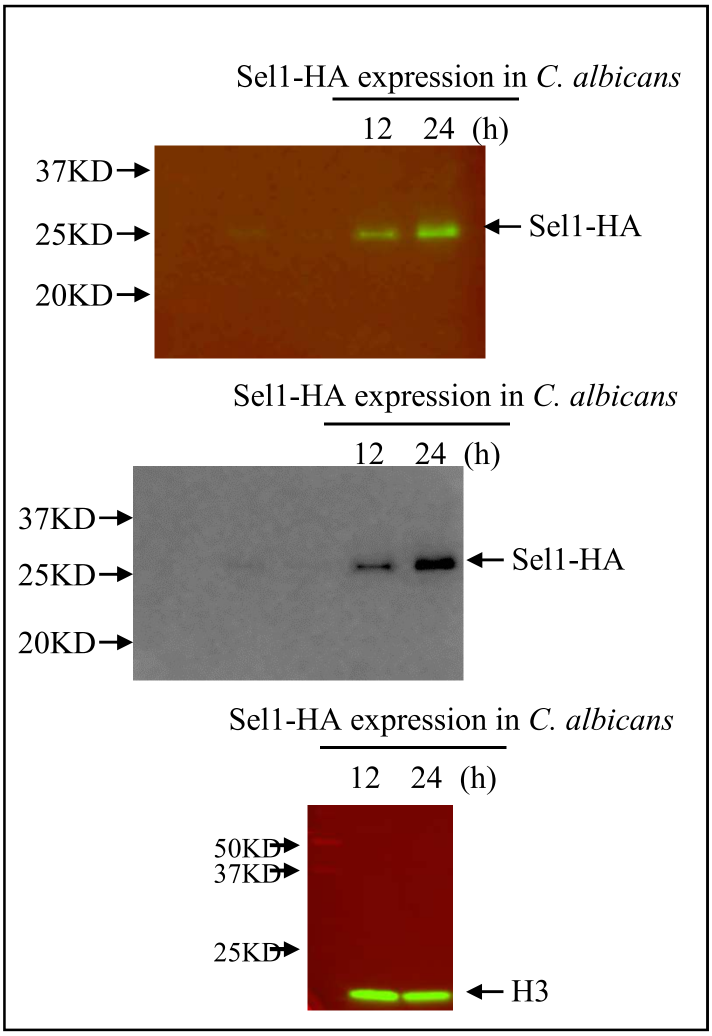

Fig. 1h

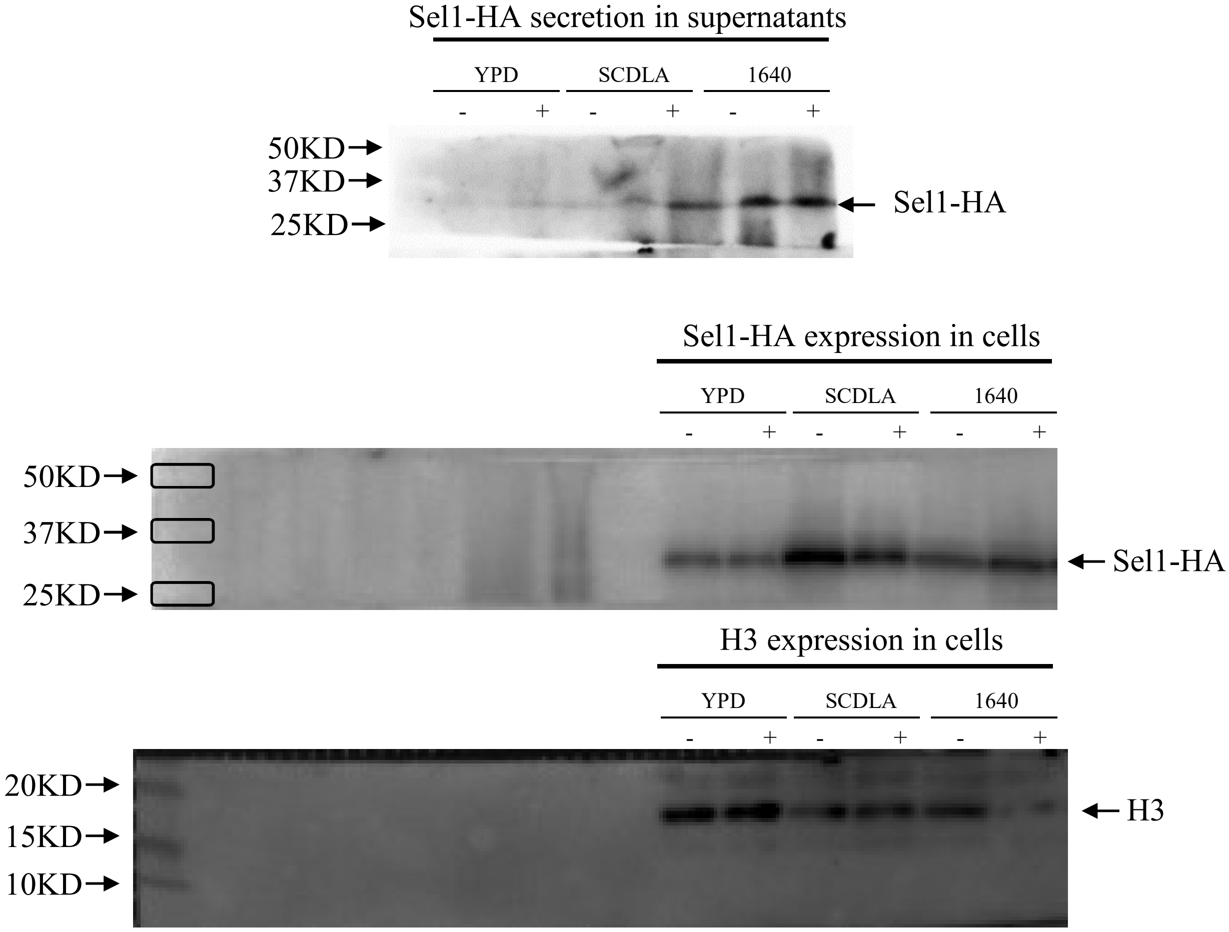

Sel1-HA secretion in supernatants
YPD
SCDLA
1640
- + - + - +
50KD
37KD
25KD
Sel1-HA
Sel1-HA expression in cells
YPD
SCDLA
1640
- + - + - +
50KD
37KD
25KD
Sel1-HA
H3 expression in cells
YPD
SCDLA
1640
- + - + - +
20KD
15KD
10KD
H3

Fig. 2d

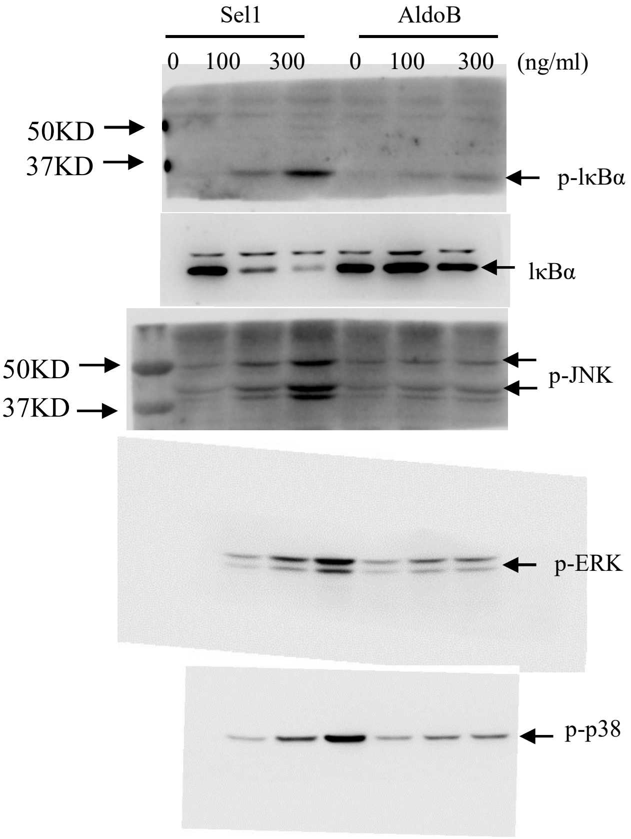
Sel1
AldoB
0 100 300 0 100 300 (ng/ml)
50KD
37KD
p-lκBα
lκBα
50KD
37KD
p-JNK
p-ERK
p-p38

Fig. 2e

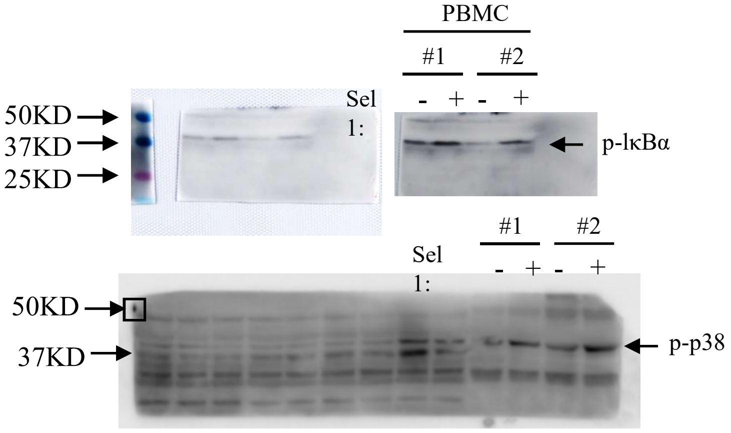
PBMC
#1 #2
- + - +
Sel 1:
50KD
37KD
25KD
p-lκBα
#1 #2
Sel 1:
- + - +
50KD
37KD
p-p38

Fig. 3f

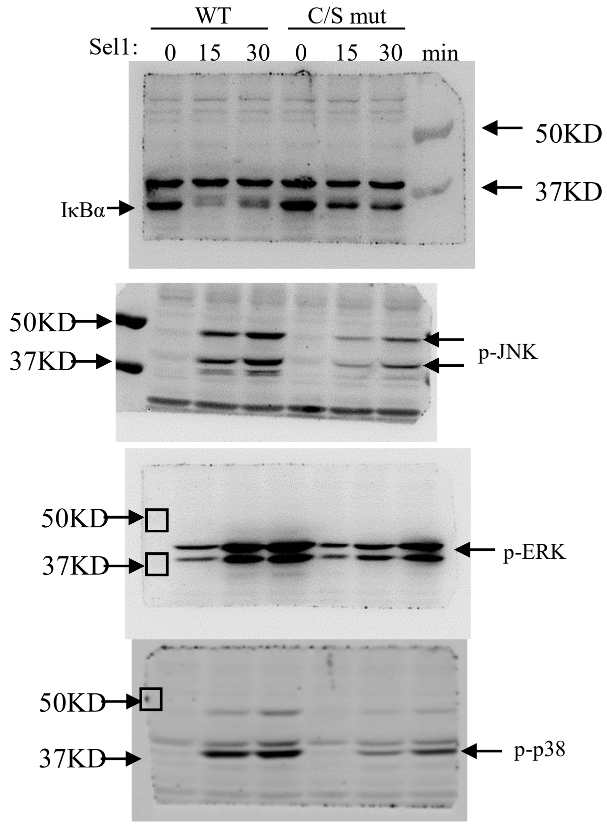
WT
C/S mut
Sel1: 0 15 30 0 15 30 min
50KD
37KD
IκBα
p-JNK
p-ERK
p-p38

Fig. 3h

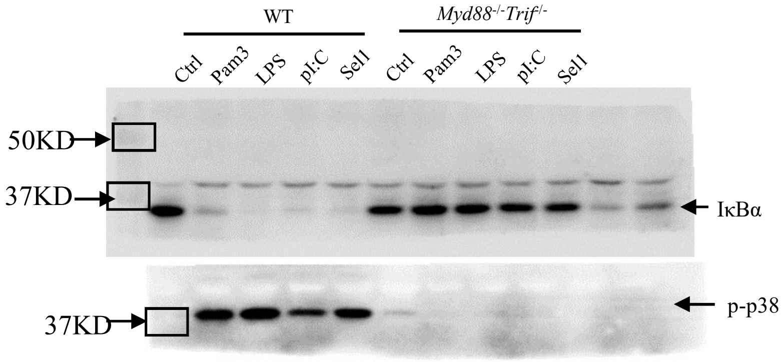
WT
Myd88-/-Trif-/-
Ctrl Pam3 LPS pI:C Sel1 Ctrl Pam3 LPS pI:C Sel1
50KD
37KD
IκBα
p-p38

Fig. 4d

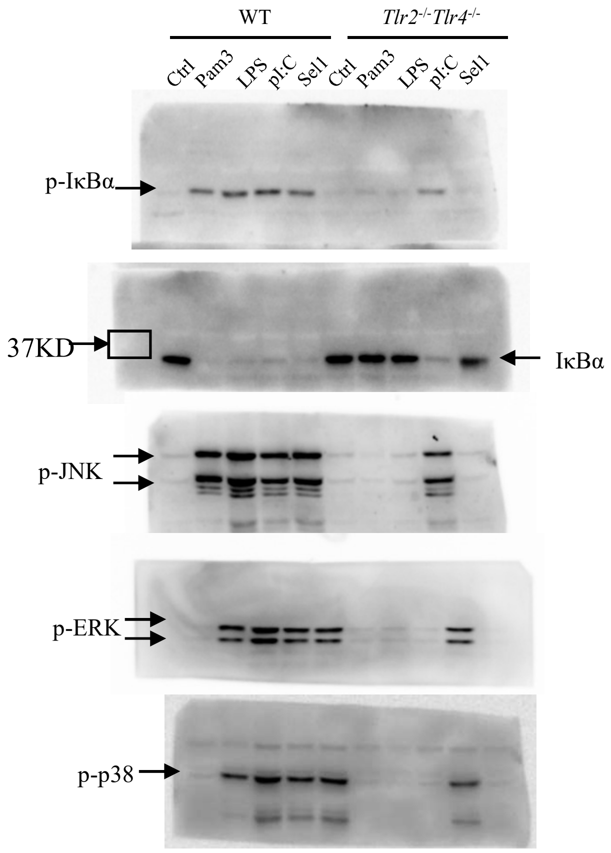

WT
Tlr2-/-Tlr4-/-
Ctrl
Pam3
LPS
pI:C
Sell
Ctrl
Pam3
LPS
pI:C
Sell
p-IκBα
37KD
IκBα
p-JNK
p-ERK
p-p38

Fig. 4e

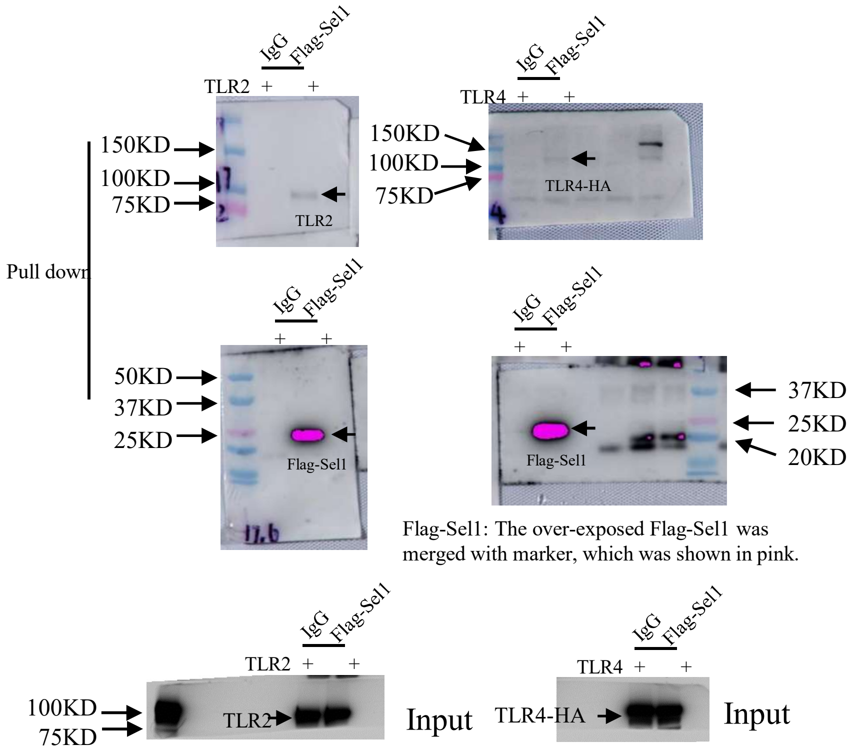

IgG
Flag-Sell
TLR2 + +
TLR4 + +
150KD
100KD
75KD
TLR2
TLR4-HA
Pull down
50KD
37KD
25KD
20KD
Flag-Sell
Flag-Sell: The over-exposed Flag-Sell was merged with marker, which was shown in pink.
Input
TLR2
TLR4-HA

Supplementary Figure 2a

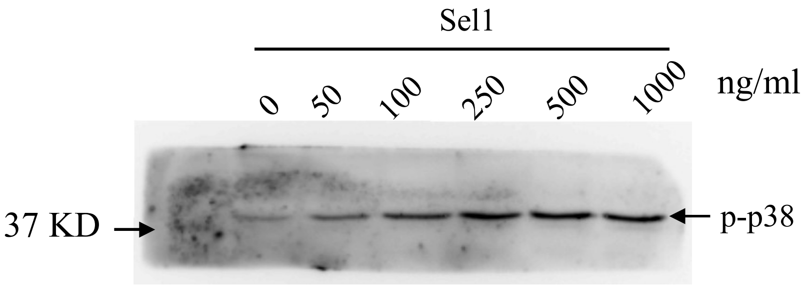

Supplementary Figure 2b

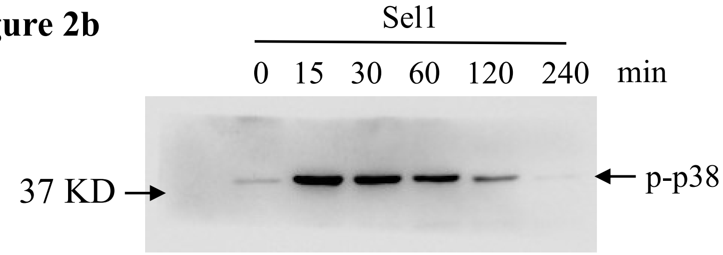

Supplementary Figure 2e

Sel1	-	+	-	+	-	+	-	+	-	+
DNase Ⅰ	-	-	+	+	-	-	-	-	-	-
RNase A	-	-	-	-	+	+	-	-	-	-
Proteinase K	-	-	-	-	-	-	+	+	-	-
PMB	-	-	-	-	-	-	-	-	+	+

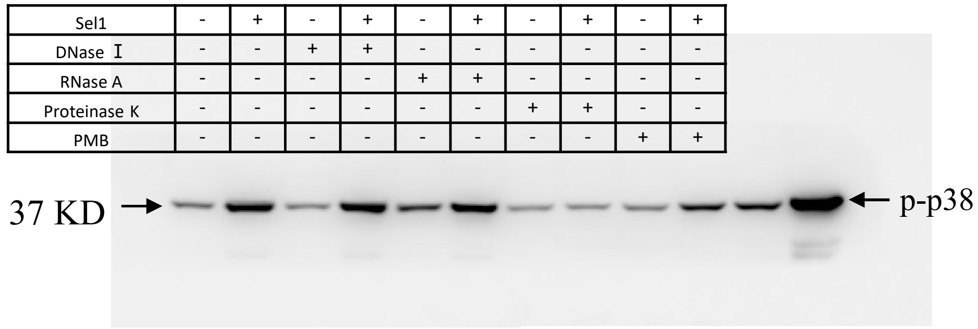

Supplementary Figure 3a

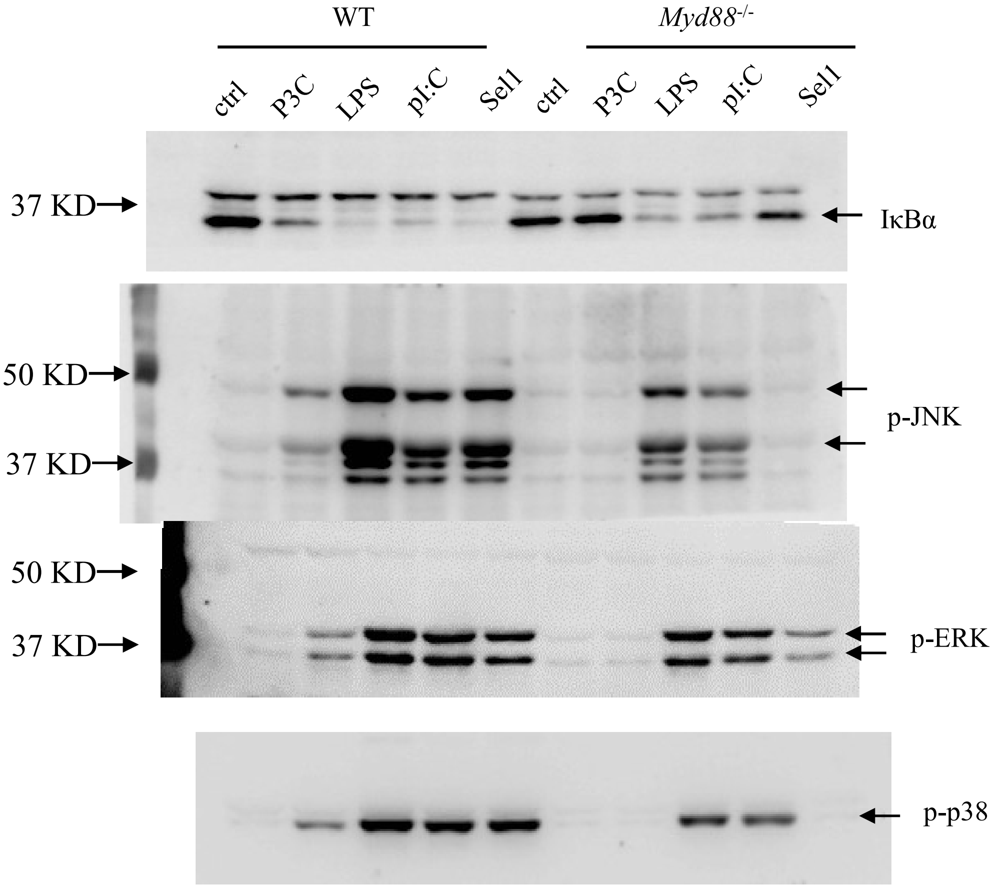

Supplementary Figure 3b

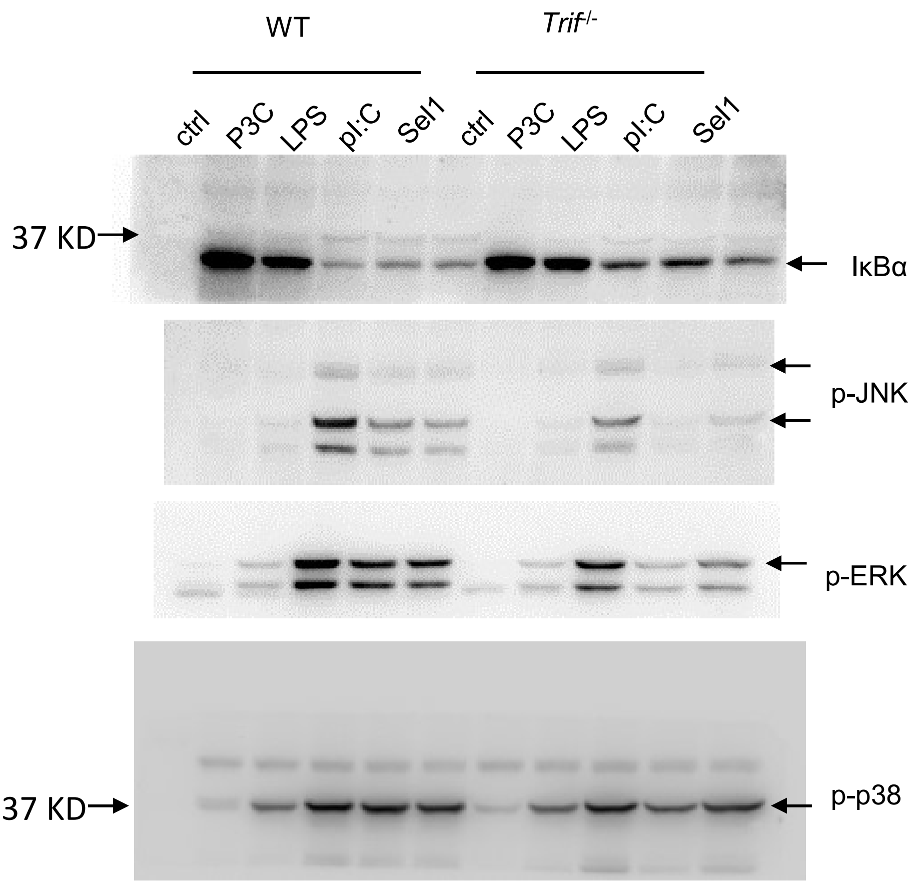

Supplementary Figure 3c

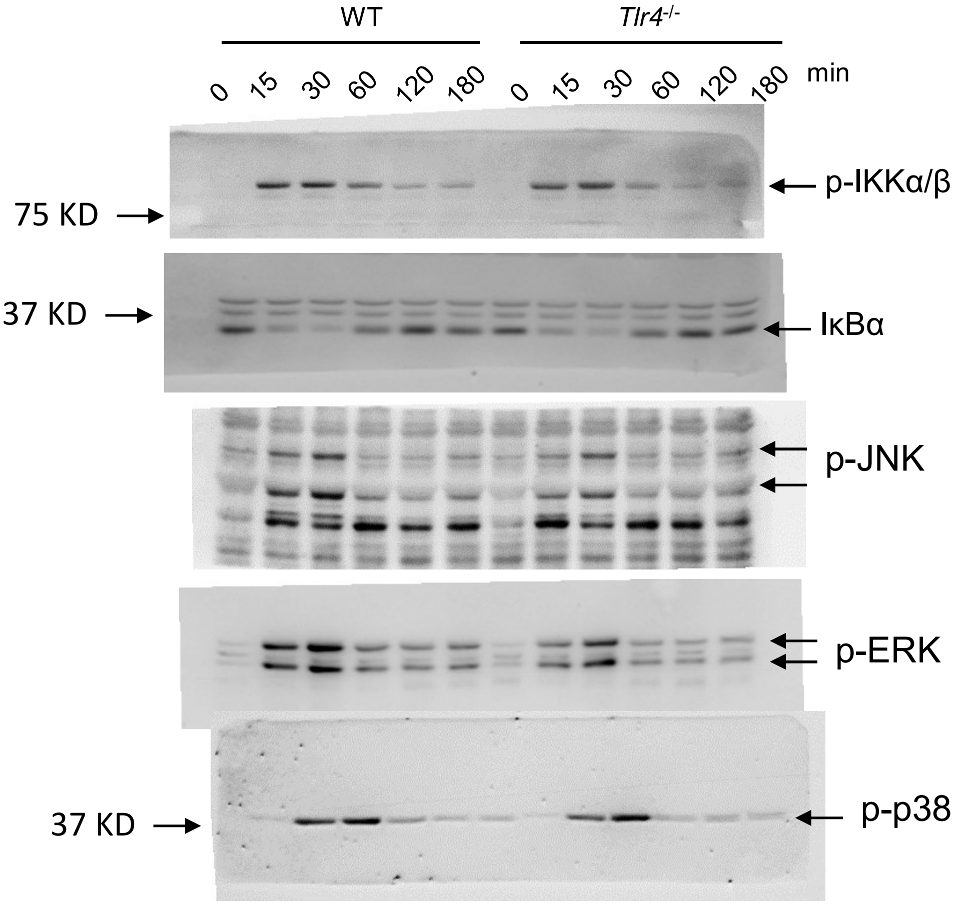

Supplementary Figure 3d

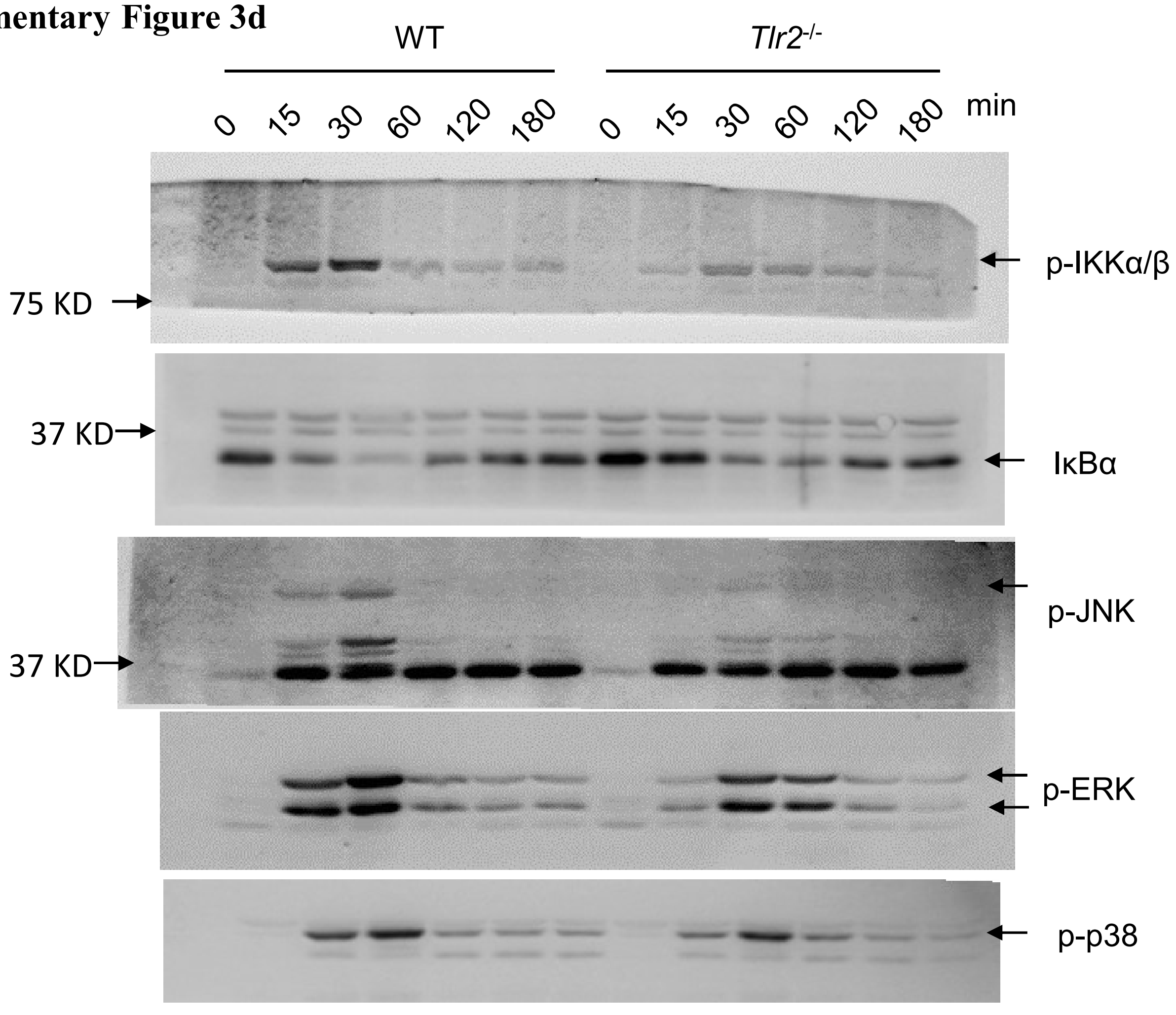

Supplementary Figure 3f

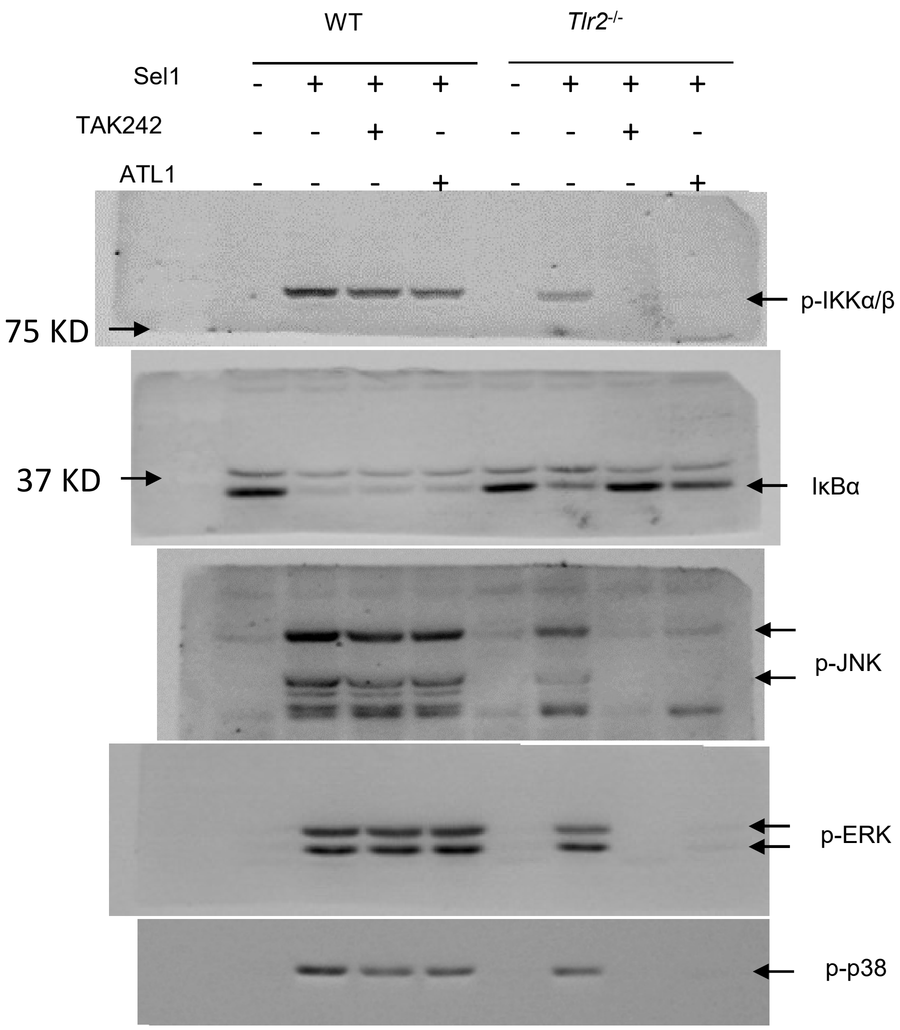

Supplement: Supplementary file 1 — Supplementary Information [file 41467_2019_8950_MOESM1_ESM.pdf]
