# Supplementary material for: A small secreted protein triggers a TLR2/4-dependent inflammatory response during invasive Candida albicans infection
Source: Nat Commun. 2019 Mar 4;10:1015. doi: 10.1038/s41467-019-08950-3 (PMC6399272; doi:10.1038/s41467-019-08950-3)
Supplement: Supplementary file 2 — Reporting Summary [file 41467_2019_8950_MOESM2_ESM.pdf]

nature research

Corresponding author(s): DBPR NCOMMS-18-18721A

Last updated by author(s): Jan 3, 2019

Reporting Summary

Nature Research wishes to improve the reproducibility of the work that we publish. This form provides structure for consistency and transparency in reporting. For further information on Nature Research policies, see Authors & Referees and the Editorial Policy Checklist.

Statistics

For all statistical analyses, confirm that the following items are present in the figure legend, table legend, main text, or Methods section.

n/a	Confirmed	
☐	☒	The exact sample size (*n*) for each experimental group/condition, given as a discrete number and unit of measurement
☐	☒	A statement on whether measurements were taken from distinct samples or whether the same sample was measured repeatedly
☐	☒	The statistical test(s) used AND whether they are one- or two-sided<br>*Only common tests should be described solely by name; describe more complex techniques in the Methods section.*
☐	☒	A description of all covariates tested
☐	☒	A description of any assumptions or corrections, such as tests of normality and adjustment for multiple comparisons
☐	☒	A full description of the statistical parameters including central tendency (e.g. means) or other basic estimates (e.g. regression coefficient) AND variation (e.g. standard deviation) or associated estimates of uncertainty (e.g. confidence intervals)
☐	☒	For null hypothesis testing, the test statistic (e.g. *F*, *t*, *r*) with confidence intervals, effect sizes, degrees of freedom and *P* value noted<br>*Give P values as exact values whenever suitable.*
☐	☒	For Bayesian analysis, information on the choice of priors and Markov chain Monte Carlo settings
☐	☒	For hierarchical and complex designs, identification of the appropriate level for tests and full reporting of outcomes
☐	☒	Estimates of effect sizes (e.g. Cohen's *d*, Pearson's *r*), indicating how they were calculated

Our web collection on statistics for biologists contains articles on many of the points above.

Software and code

Policy information about availability of computer code

Data collection	No software was used.
Data analysis	No software was used.

For manuscripts utilizing custom algorithms or software that are central to the research but not yet described in published literature, software must be made available to editors/reviewers. We strongly encourage code deposition in a community repository (e.g. GitHub). See the Nature Research guidelines for submitting code & software for further information.

Data

Policy information about availability of data

All manuscripts must include a data availability statement. This statement should provide the following information, where applicable:

- Accession codes, unique identifiers, or web links for publicly available datasets
- A list of figures that have associated raw data
- A description of any restrictions on data availability

The authors declare that the main data supporting the findings of this study are available within the article and its Supplementary Information. Extra data that support the findings of this study are available from the corresponding authors upon reasonable request.

Field-specific reporting

Please select the one below that is the best fit for your research. If you are not sure, read the appropriate sections before making your selection.

☒ Life sciences ☐ Behavioural & social sciences ☐ Ecological, evolutionary & environmental sciences

Life sciences study design

All studies must disclose on these points even when the disclosure is negative.

Sample size	All the experiments have been described in the Methods section.
Data exclusions	No data were excluded from the analyses.
Replication	The reproducibility of the experiments have been described in the figure legends.
Randomization	Yes, we are.
Blinding	Yes, we are.

Reporting for specific materials, systems and methods

We require information from authors about some types of materials, experimental systems and methods used in many studies. Here, indicate whether each material, system or method listed is relevant to your study. If you are not sure if a list item applies to your research, read the appropriate section before selecting a response.

Materials & experimental systems

n/a	Involved in the study	
☐	☒	Antibodies
☐	☒	Eukaryotic cell lines
☒	☐	Palaeontology
☐	☒	Animals and other organisms
☒	☐	Human research participants
☒	☐	Clinical data

Methods

n/a	Involved in the study	
☒	☐	ChIP-seq
☒	☐	Flow cytometry
☒	☐	MRI-based neuroimaging

Antibodies

Antibodies used	p-IkBa, Cell signaling Technology, Cat#2859; p-p38, Cell signaling Technology, Cat#9211; p-JNK, Cell Signaling Technology, Cat#4668;JNK, Cell Signaling Technology, Cat#9252;p-IKKa/b, Cell Signaling Technology, Cat#2697;p-Syk, Cell Signaling Technology, Cat#2710;IkBa, Santa Cruz Biotechnology, SC-1643;p38, Santa Cruz Biotechnology, SC-728;p-ERK, Santa Cruz Biotechnology, SC-7383;ERK, Santa Cruz Biotechnology, SC-93;HA, Sigma-Aldrich, H6908;H3, Abcams, ab-1791;b-ACTIN, Abways technology, ab2001;
Validation	All the antibodies used in this study are commercial.

Eukaryotic cell lines

Policy information about cell lines

Cell line source(s)	All the cell lines used are from ATCC. Raw 264.7 cells (ATCC® TIB-71); L929 cells (ATCC® CRL-6364); J558L cells (ATCC® TIB-6)
Authentication	All the cell lines are authorized.
Mycoplasma contamination	All cell lines tested negative of mycoplasma contamination.
Commonly misidentified lines (See ICLAC register)	No commonly misidentified cell lines are used.

Animals and other organisms

Policy information about studies involving animals; ARRIVE guidelines recommended for reporting animal research

Laboratory animals	The ICR (18-21g, male) and C57BL/6 (6-8 week, male) wild type mice were purchased from Shanghai Laboratory Animal Center (SLAC). The immune deficient mice (Myd88-/-, Trif-/-, Tlr2-/-, Tlr4-/-, Tlr5-/-, Tlr7-/-, Il1r-/-) were obtained from Jackson Labs. The Tlr2-/-Tlr4-/- and Myd88-/-Trif-/- mice were generated in this study. All the genes knockout mice were used when they are 6-10 weeks old. The experiments were conducted in individual ventilated cages in a pathogen-free facility at Shanghai Institute of Biochemistry and Cell Biology following a protocol approved by the Institutional Animal Care and Use Committee of the Shanghai Institutes for Biological Sciences, Chinese Academy of Sciences.

Wild animals	The study did not involve wild animals.
Field-collected samples	The study did not involve samples collected from the field.
Ethics oversight	All the animals experiments were approved by the Institutional Animal Care and Use Committee of the Shanghai Institutes for Biological Sciences, Chinese Academy of Sciences.

Note that full information on the approval of the study protocol must also be provided in the manuscript.
